# Disrupted myelin lipid metabolism differentiates frontotemporal dementia caused by *GRN* and *C9orf72* gene mutations

**DOI:** 10.1186/s40478-023-01544-7

**Published:** 2023-03-27

**Authors:** Oana C. Marian, Jonathan D. Teo, Jun Yup Lee, Huitong Song, John B. Kwok, Ramon Landin-Romero, Glenda Halliday, Anthony S. Don

**Affiliations:** 1 Charles Perkins Centre, The University of Sydney, Camperdown, NSW 2006 Australia; 2 School of Medical Sciences, The University of Sydney, Camperdown, NSW 2006 Australia; 3 Brain and Mind Centre, The University of Sydney, Camperdown, NSW 2006 Australia; 4 School of Health Sciences, The University of Sydney, Camperdown, NSW 2006 Australia

**Keywords:** FTD, Progranulin, Lipidomics, Lysosome, Cholesterol, TDP-43

## Abstract

Heterozygous mutations in the *GRN* gene and hexanucleotide repeat expansions in *C9orf72* are the two most common genetic causes of Frontotemporal Dementia (FTD) with TDP-43 protein inclusions. The triggers for neurodegeneration in FTD with *GRN* (FTD-*GRN*) or *C9orf72* (FTD-*C9orf72*) gene abnormalities are unknown, although evidence from mouse and cell culture models suggests that *GRN* mutations disrupt lysosomal lipid catabolism. To determine how brain lipid metabolism is affected in familial FTD with TDP-43 inclusions, and how this is related to myelin and lysosomal markers, we undertook comprehensive lipidomic analysis, enzyme activity assays, and western blotting on grey and white matter samples from the heavily-affected frontal lobe and less-affected parietal lobe of FTD-*GRN* cases, FTD-*C9orf72* cases, and age-matched neurologically-normal controls. Substantial loss of myelin-enriched sphingolipids (sulfatide, galactosylceramide, sphingomyelin) and myelin proteins was observed in frontal white matter of FTD-*GRN* cases. A less-pronounced, yet statistically significant, loss of sphingolipids was also observed in FTD-*C9orf7*2. FTD-*GRN* was distinguished from FTD-*C9orf72* and control cases by increased acylcarnitines in frontal grey matter and marked accumulation of cholesterol esters in both frontal and parietal white matter, indicative of myelin break-down. Both FTD-*GRN* and FTD-*C9orf72* cases showed significantly increased lysosomal and phagocytic protein markers, however galactocerebrosidase activity, required for lysosomal catabolism of galactosylceramide and sulfatide, was selectively increased in FTD-*GRN*. We conclude that both *C9orf72* and *GRN* mutations are associated with disrupted lysosomal homeostasis and white matter lipid loss, but *GRN* mutations cause a more pronounced disruption to myelin lipid metabolism. Our findings support the hypothesis that hyperactive myelin lipid catabolism is a driver of gliosis and neurodegeneration in FTD-*GRN*. Since FTD-*GRN* is associated with white matter hyperintensities by MRI, our data provides important biochemical evidence supporting the use of MRI measures of white matter integrity in the diagnosis and management of FTD.

**Supplementary Information:**

The online version contains supplementary material available at 10.1186/s40478-023-01544-7.

## Introduction

FTD is the second most common cause of younger-onset dementia, frequently manifesting before 65 years of age [1, 2]. It is characterised by atrophy of the frontal or temporal lobes and classified into three clinical syndromes: behavioural variant FTD (bvFTD), progressive non-fluent aphasia, and semantic dementia. BvFTD, typified by personality changes, socially inappropriate behaviour and cognitive deficits, is the most common [3]. Pathologically, FTD is associated with intraneuronal aggregates of hyperphosphorylated tau protein or hyperphosphorylated and proteolytically-cleaved 43 kDa TAR DNA-binding protein (TDP-43) in both neurons and glia [4].

A strong family history is reported in nearly half of all bvFTD cases [3]. The two most common genetic causes of familial FTD with TDP-43 inclusions are heterozygous mutations in the gene encoding progranulin (*GRN*), which accounts for 5–20% of inherited FTD [5, 6]; and hexanucleotide repeat (GGGGCC) expansions in a non-coding region of the *C9orf72* gene, accounting for approximately 20% of inherited FTD [7]. *C9orf72* repeat expansions are also the most common genetic cause of amyotrophic lateral sclerosis (ALS) [8].

Progranulin is a secreted glycoprotein that is highly expressed by activated microglia [6, 9], interacts with the neuronal receptor sortilin, and localises primarily to neuronal and microglial lysosomes [9, 10]. Heterozygous *GRN* mutations that cause FTD lead to nonsense-mediated decay of *GRN* mRNA and reduced progranulin protein levels [5, 6]. Loss of function mutations in both *GRN* alleles cause the severe lysosomal storage disorder neuronal ceroid lipofuscinosis (NCL), characterised by enlarged lysosomes and accumulation of the auto-fluorescent material lipofuscin [11, 12]. These features are also observed in brains of FTD-*GRN* cases and *Grn*-/- mice [13–16]. Mechanistic studies have demonstrated important functions for progranulin in regulating lysosomal acidification [17], the lysosomal import of proteins required for lipid catabolism [10], and activity of the lysosomal lipase glucocerebrosidase (GCase) [18, 19].

*C9orf72* repeat expansions are associated with reduced *C9orf72* transcript levels, nuclear RNA foci that interfere with the function of RNA binding proteins, and inclusions comprising dipeptide repeat polymers derived from translation of the repeat expansions [8, 20, 21]. Whether one of these mechanisms predominates in causing neurodegeneration remains a subject of research, since both loss of normal *C9orf72* and gain of toxic function promote neurodegenerative phenotypes [22, 23]. Physiologically, C9orf72 is required for endosomal trafficking, autophagy and lysosomal biogenesis [23, 24]. Disrupted lysosomal function may therefore be common to both FTD-*GRN* and FTD-*C9orf72*.

Magnetic resonance imaging (MRI) studies show white matter hyperintensities, indicative of focal demyelination, in FTD-*GRN* but not FTD-*C9orf72* cases [25–27]. On the other hand, diffusion tensor imaging has shown reduced white matter integrity in both FTD-*GRN* and FTD-*C9orf72*, and in bvFTD more generally [28, 29]. Myelin is composed 70% (dry weight) of lipids [30]. The physiological turnover and maintenance of myelin is therefore predicted to require constitutive lipid catabolism in microglial and oligodendrocyte lysosomes. In this study we demonstrate that both FTD-*GRN* and FTD-*C9orf72* are characterised by significant lipid loss in frontal white matter. However, myelin lipid and protein loss was substantially greater in FTD-*GRN* cases and associated with a pronounced increase in cholesterol esters, suggesting that excess cholesterol and fatty acids resulting from myelin break-down are stored as cholesterol esters in white matter. Markers of phagocytic microglia, TREM2 and CD68, were increased in FTD-*C9orf72* and FTD-*GRN*, indicating that both gene defects promote a phagocytic microglial phenotype, however our evidence indicates that hyperactive myelin lipid catabolism differentiates FTD-*GRN* from FTD-*C9orf72* cases.

## Materials and methods

### Human brain tissue

Fresh frozen grey and white matter tissue samples from the superior frontal and superior parietal lobe of 11 FTD-*C9orf72* cases, 6 FTD-*GRN* cases, and 11 age-matched neurologically-normal controls were obtained from the Sydney Brain Bank and NSW Brain Tissue Resource Centre. Demographic and clinical information (sex, age at death, cause of death and post-mortem interval (PMI)) is provided in Table 1.

**Table 1:** Demographic information for cases used in this study. PMI: post-mortem interval. *Cases used for histological analysis. ^#^Cases with co-occurring ALS.

Case ID	Gene Defect	Age​	Sex​	PMI (h)​	Tissue pH​	Cause of Death​	TDP-43 Type​
1​	C9orf72​	65​	F​	5​	6.6​	Bronchopneumonia​	A​
2​	C9orf72​	49​	F​	26​	6.3​	Aspiration pneumonia​	B​
3​	C9orf72​	66​	M​	9​	6.2	Cardiorespiratory failure​	B​
4​	C9orf72​	81​	F​	14​	6.6​	Cardiorespiratory arrest​	B​
5^#^​	C9orf72​	68​	F​	9​	6.3​	Cardiorespiratory failure​	A​
6​	C9orf72​	75​	F​	46​	6.3​	Cardiorespiratory failure​	A​
7​^#^	C9orf72​	70​	M​	15​	6.4​	Cardiorespiratory failure​	A​
8*​	C9orf72​	61​	M​	39​	6.3​	Aspiration pneumonia​	A​
9*​	C9orf72​	69​	F​	24​	5.6​	Cardiorespiratory failure​	B​
10*​	C9orf72​	67​	F​	22​	6.2​	Cardiorespiratory failure​	A​
11*​	C9orf72​	83​	M​	13​	5.9​	Uraemia, bronchopneumonia​	A​
12​	GRN (c.90_91insCTGS)	77​	M​	48​	6.2​	Aspiration pneumonia​	A​
13*​	GRN​ ​(c.90_91insCTGS)	54​	F​	26​	5.9​	Aspiration pneumonia​	A​
14*​	GRN​ (c.898 C > T)	54​	F​	21​	5.8​	Cardiorespiratory failure​	A​
15​	GRN​ (c.898 C > T)	61​	M​	17​	6.0​	Cardiorespiratory failure​	A​
16​	GRN​ (c.87dup)	64​	M​	13​	5.6​	Cardiorespiratory failure​	A​
17*​	GRN​ (c.918 C > A)	68​	F​	29​	6.1​	Cardiorespiratory failure​	A​
18*​	Control​	69​	M​	16​	6.6​	Cardiac​	-​
19*​	Control​	60​	M​	25​	6.7​	Infection​	-​
20*​	Control​	71​	F​	16​	6.2​	Cancer​	-​
21​	Control​	66​	M​	23​	6.7​	Cardiac​	-​
22​	Control​	66​	M​	63​	6.9​	Cardiac​	-​
23​	Control​	69​	F​	39​	6.7​	Cardiac/ Respiratory​	-​
24​	Control​	73​	M​	9​	6.5​	Cancer​	-​
25​	Control​	51​	F​	41​	7.0​	Alcohol toxicity​	-​
26​	Control​	84​	M​	36​	6.4​	Severe pulmonary hypertension​	-​
27*​	Control​	80​	F​	29​	6.3​	Cardiorespiratory failure​	-​
28*​	Control​	84​	F​	16​	5.7​	Endocarditis​	-​

Approximately 100 mg of frozen brain tissue was homogenised for 1 min at 4 °C in 700 µL ice-cold HEPES buffer (50 mM, pH 7.4) containing 5 mM NaF, 2 mM Na_3_VO_4_, 10 mM KCl and cOmplete Mini EDTA-free Protease Inhibitor Cocktail (Roche #11836153001), using a bead beater with 425–600 mm acid washed glass beads (Sigma Aldrich #G8772). Samples were centrifuged at 4 °C for 1 min at 1000x*g* and the homogenate was transferred to a new tube, after which the beads were washed with a further 100 µL of ice-cold HEPES buffer, centrifuged, and the supernatant combined with the previous fraction. The homogenate was stored in 100 µL aliquots at -80 °C. Protein concentration of the homogenates was determined by bicinchoninic acid assay (Thermo Scientific #23225).

### Lipid extraction

Lipids were extracted from 100 µL brain homogenate samples (~ 100 µg protein) using a two-phase methyl-tert-butyl ether (MTBE)/methanol/water protocol [31]. Homogenate was combined with 850 µL MTBE and 250 µL methanol containing internal standards: 5 nmoles PC(19:0/19:0); 2 nmoles each of SM(d18:1/12:0), GluCer(d18:1/12:0), PS(17:0/17:0), PE(17:0/17:0), PG(17:0/17:0), CL(14:0/14:0/14:0/14:0), TG(17:0/17:0/17:0), and CholE(17:0); 1 nmole PA(17:0/17:0), PI(d7-18:1/15:0), and d7-Chol; 0.5 nmoles LacCer(d18:1/12:0), ST(d18:1/17:0), Cer(d18:1/17:0), DG(d7-18:1/15:0), MG(d7-18:1), LPC(17:0), LPE(17:1), LPS(17:1); and 0.2 nmoles Sph(d17:1), S1P(d17:1), LPA(17:0), and AcCa(d3-16:0). Samples were sonicated in a 4 °C water bath for 30 min. Phase separation was induced with the addition of 212 µL of mass spectrometry grade water, samples were vortexed and centrifuged at 2000x*g* for 5 min and the upper organic phase was collected in 5 mL glass tubes. The aqueous phase was extracted twice more with 500 µL MTBE and 150 µL methanol followed by sonication for 15 min and phase separation with 125 µL water. Organic phases from the three extractions were combined and dried under vacuum in a Savant SC210 SpeedVac (ThermoFisher Scientific). Lipids were reconstituted in 400 µL of HPLC grade methanol, then diluted 1:5 in 80% (v/v) methanol:20% water containing 1 mM ammonium formate and 0.2% formic acid.

### Lipid quantification using liquid chromatography-tandem mass spectrometry (LC-MS/MS)

Lipidomic data was acquired with a ThermoFisher Q-Exactive HF-X mass spectrometer coupled to a Vanquish HPLC [31]. Lipids were resolved on a 2.1 × 100 mm Waters C18 HPLC column (1.7 μm pore size), using a 27 min binary gradient at a 0.28 mL/minute flow rate: 0 min, 80:20 A/B; 3 min, 80:20 A/B; 5.5 min, 55:45 A/B; 8 min, 36:65 A/B; 13 min, 15:85 A/B; 14 min, 0:100 A/B; 20 min, 0:100 A/B; 20.2 min, 70:30 A/B; 27 min, 70:30 A/B. Solvent A was 10 mM ammonium formate, 0.1% formic acid in acetonitrile:water (60:40); Solvent B was 10 mM ammonium formate, 0.1% formic acid in isopropanol:acetonitrile (90:10). Data was acquired in full scan/data-dependent MS^2^ mode (resolution 60,000 FWHM, scan range 220–1600 *m/z*). Sample order was randomised, and data was collected in both positive and negative mode for each sample. The ten most abundant ions in each cycle were subjected to MS^2^, with an isolation window of 1.4 *m/z*, collision energy 30 eV, resolution 17,500 FWHM, maximum integration time 110 ms and dynamic exclusion window 10 s. An exclusion list of background ions was based on a solvent blank. An inclusion list of the [M + H]^+^ and [M-H]^−^ ions for all internal standards was used. Mass accuracy was < 5 ppm and %CV for peak elution time was < 2% across the entire sample run.

LipidSearch software (version 4.2, Thermo Fisher) was used for lipid annotation, chromatogram alignment, and peak integration. Lipid annotation required both accurate precursor ion mass (5 ppm mass tolerance) and diagnostic product ions (8 ppm mass tolerance). Molar amounts for each lipid were calculated by taking the ratio to the class-specific internal standard, after which lipid levels were normalised to protein content.

### Bis(monoacylglycero)phosphate (BMP) quantification

Resolution of BMP from its mass isomer PG was performed as described [19]. Lipid annotation, chromatogram alignment and peak integration were carried out with TraceFinder software (version 5.1, Thermo Fisher). Correct peak identification was confirmed with the use of commercial standards for BMP(18:1/18:1) and PG(18:1/18:1) (Avanti Polar Lipids #857135 and #840475, respectively). Molar amounts for BMP and PG were calculated relative to the PG(17:0/17:0) internal standard.

### Western blots

Whole brain homogenates (10 µg protein for grey matter and 5 µg for white matter) were resolved on Bolt™ 4–12% Bis-Tris Plus gels (ThermoFisher Scientific #NW04125BOX) and transferred to polyvinylidene fluoride membranes. Membranes were blocked for 1 h at RT with 5% skim milk in Tris-buffered saline containing 0.1% Tween-20 (TBST), then incubated overnight at 4 °C with primary antibody in TBST with 3% bovine serum albumin (Sigma Aldrich #A7906). Membranes were then washed 3 times in TBST and incubated in horseradish peroxidase-conjugated secondary antibody diluted 1:5000 in TBST containing 5% skim milk for 2 h at RT. Membranes were imaged with ECL Ultra Western HRP Substrate (Millipore #WBULS0500) using a Bio-Rad ChemiDoc Touch. Bands were quantified by densitometry with Bio-Rad Image Lab software (v6.0.1). Membranes were then stripped with mild stripping buffer (1.5% w/v glycine, 0.1% w/v SDS, 0.1% v/v Tween20, pH 2.2), blocked with 5% skim milk in TBST for 1 h and re-probed with anti-β-actin (Abcam #ab8227, RRID #AB_2305186) or anti-GAPDH (Cell Signalling #2118, RRID #AB_561053) at 1:5000 dilution in TBST with 3% BSA overnight at 4 °C. A common sample was included on each gel as a loading control to normalise between membranes containing different samples.

The following primary antibodies were used at 1:1000 dilution unless specified: rabbit anti-MBP (Abcam, #ab40390, RRID #AB_1141521), rabbit anti-PLP (Abcam #ab28486, RRID #AB_776593) (diluted 1:2000), mouse anti-CNP (11-5B) (Abcam #ab6319, RRID #AB_2082593), mouse anti-NEFL (DA2) (Invitrogen #13–0400, RRID #AB_2532995), mouse anti-tubulin βIII (TUBB3) (BioLegend #801,202, RRID #AB_10063408) (diluted 1:5000), #rabbit anti-LAMP1 (D2D11) XP® (Cell Signalling #9091, RRID #AB_2687579), mouse anti-LAMP2 (Developmental Studies Hybridoma Bank #H4B4, RRID #AB_528129) (diluted 1:200), rabbit anti-Trem2 (Cell Signalling #91,068, RRID # AB_1961900), rabbit anti-CD68 (Abcam # ab213363, RRID #AB_2801637).

### Enzyme activity assays

β-glucocerebrosidase (GCase) and β-galactocerebrosidase (GALC) activities were assayed with the fluorometric substrates 4-Methylumbelliferyl β-D-glucopyranoside (Sigma-Aldrich #M3633) and 4-Methylumbelliferyl β-D-galactopyranoside (Sigma-Aldrich #M1633), respectively. All reactions were carried out in triplicate in 96-well white Opti-Plates (PerkinElmer #6005290). For GCase activity, 1 µg homogenate protein was resuspended in 15 µL 0.1 M citric acid/0.2 M disodium phosphate (pH 5) and incubated with 30 µL of 10 mM substrate dissolved in 0.1 M citric acid/0.2 M disodium phosphate (pH 5), 0.5% sodium taurocholate, 0.25% Triton X-100 [32]. Plates were covered with sealing film, shaken, and incubated at 37 °C in the dark for 1 h. Reactions were stopped with 180 µL of ice-cold stop solution (0.2 M glycine/NaOH, pH 10.4). For GALC activity, 20 µg homogenate protein was resuspended in 25 µL citrate/phosphate buffer, pH 4.5, and incubated for 30 min with 25 µL of 1 mM substrate dissolved in 50 mM sodium citrate, pH 4.5, 125 mM NaCl, 0.5% Triton X-100. Reactions were stopped with 50 µL of ice-cold stop solution (0.5 M glycine/0.3 M NaOH, pH 10). Fluorescence was measured on a Tecan M200 Pro plate reader with excitation 360 nm and emission 446 nm. Relative activity was determined after subtraction of the substrate blank.

### Immunohistochemistry

TDP-43 inclusions were detected with anti-phospho TDP-43 (pS409/410) (Cosmo Bio, CAC-TIP-PTD-M01, RRID # AB_1961900). The type and density of TDP-43 inclusions and dystrophic neurites was assessed by an experienced research neuropathologist.

Formalin-fixed and paraffin embedded Sect. (10 μm) from the superior frontal lobe of 4 FTD-*C9orf72* cases, 3 FTD-*GRN* cases and 5 age-matched controls, indicated by an asterisk in Table 1, were used for luxol fast blue (LFB) staining, and immunofluorescence staining for aspartoacylase (ASPA) and myelin basic protein (MBP). These cases were those for which the tissue had not been subjected to extended fixation (> 2 weeks). Sections were heated in an oven at 60 °C for 1 h, deparaffinised in 2 × 15 min changes of xylene, and rehydrated to deionised water following graded changes of ethanol from 100 to 50%. For LFB staining, sections were rehydrated to 95% ethanol, incubated in 0.1% LFB solution (#S3382, Sigma-Aldrich) at 60 °C for 17 h, then rinsed in 70% ethanol for 2.5 min, followed by distilled water until the water ran clear. Sections were then differentiated in 0.05% lithium carbonate solution for 30 s, then in 70% ethanol for 30 s, and rinsed in distilled water. Sections were then counterstained with 0.1% cresyl violet solution for 30 s and rinsed in distilled water, followed by 95% ethanol for 5 min. Sections were dehydrated in two changes of 100% ethanol, cleared in two changes of xylene, and coverslipped using DPX mounting medium. Myelination scores from 0 to 3 were assigned by two blinded observers, where 0 is complete absence of myelin and 3 is dense myelin [33].

For immunofluorescence, sections were incubated in sodium citrate antigen retrieval buffer (10 mM, pH 6.0, 0.05% Tween 20) at 85 °C for 10 min, blocked in PBS with 0.1% Triton X-100 (PBST), 5% normal goat serum and 0.1% bovine serum albumin (BSA) at RT for 2 h, and incubated overnight at 4 °C with primary antibodies rabbit anti-ASPA (Abcam #ab223269 EPR22072) and mouse anti-MBP (R&D #MAB42282), diluted 1:250 in blocking solution. Sections were incubated in secondary antibodies (AlexaFluor 488 Goat anti-mouse, Cell Signalling #4408, RRID: AB_10694704; and AlexaFluor 647 Goat anti-rabbit, Cell Signalling #4414, RRID: AB_10693544) diluted 1:250 in blocking solution for 2 h, and counterstained with 1 µg/mL diamidino-2-phenylindole dihydrochloride (DAPI). Autofluorescence was quenched with TrueBlack Plus autofluorescence eliminator (Biotium #23,014) per manufacturer’s protocol. Sections were cover-slipped with ProLong Glass antifade mountant (Life Technologies #P36980) before imaging. Slides were imaged with a Zeiss Axioscan slide scanner. The density of ASPA-positive cells (co-localised ASPA and DAPI staining) in white matter was quantified using QuPath (version 0.3.2) [34].

### Statistical analysis

Partial least squares-discriminant analysis (PLS-DA) of lipidomic data was carried out in MetaboAnalyst (version 5.0). Lipid levels were log_10_-transformed and filtered based on interquartile range prior to analysis. Missing values (not detected in that sample) were assigned a value of 1/5 of the minimum observed value for that lipid. Lipid class totals, and individual cholesterol ester or acylcarnitine species, were compared between the three sample groups using one-way ANOVA adjusted for age and PMI, followed by Tukey’s post-hoc test. Values were natural log-transformed to improve normality and ANOVA *p* values were adjusted for false discovery rate using the Benjamini-Hochberg correction, with adjusted *p* < 0.05 considered significant. These statistical tests were performed using the *car*, *olsrr*, *ggplot2*, *multcomp* and *dplyr* packages in R (version 4.0.3). The heatmap was generated in Tableau Desktop (version 2022.1.1).

Western blot, immunofluorescence, BMP levels, and enzyme activity data were analysed by one-way ANOVA with Tukey’s post-hoc test, using GraphPad PRISM (version 9.3.1). Non-normally distributed data were natural log-transformed to achieve a normal distribution. LFB histological scores were subject to non-parametric Kruskall-Wallis test with Dunn’s post-test. Spearman correlations were performed in GraphPad PRISM.

## Results

### Case demographics and TDP-43 pathology

**Fig. 1:**
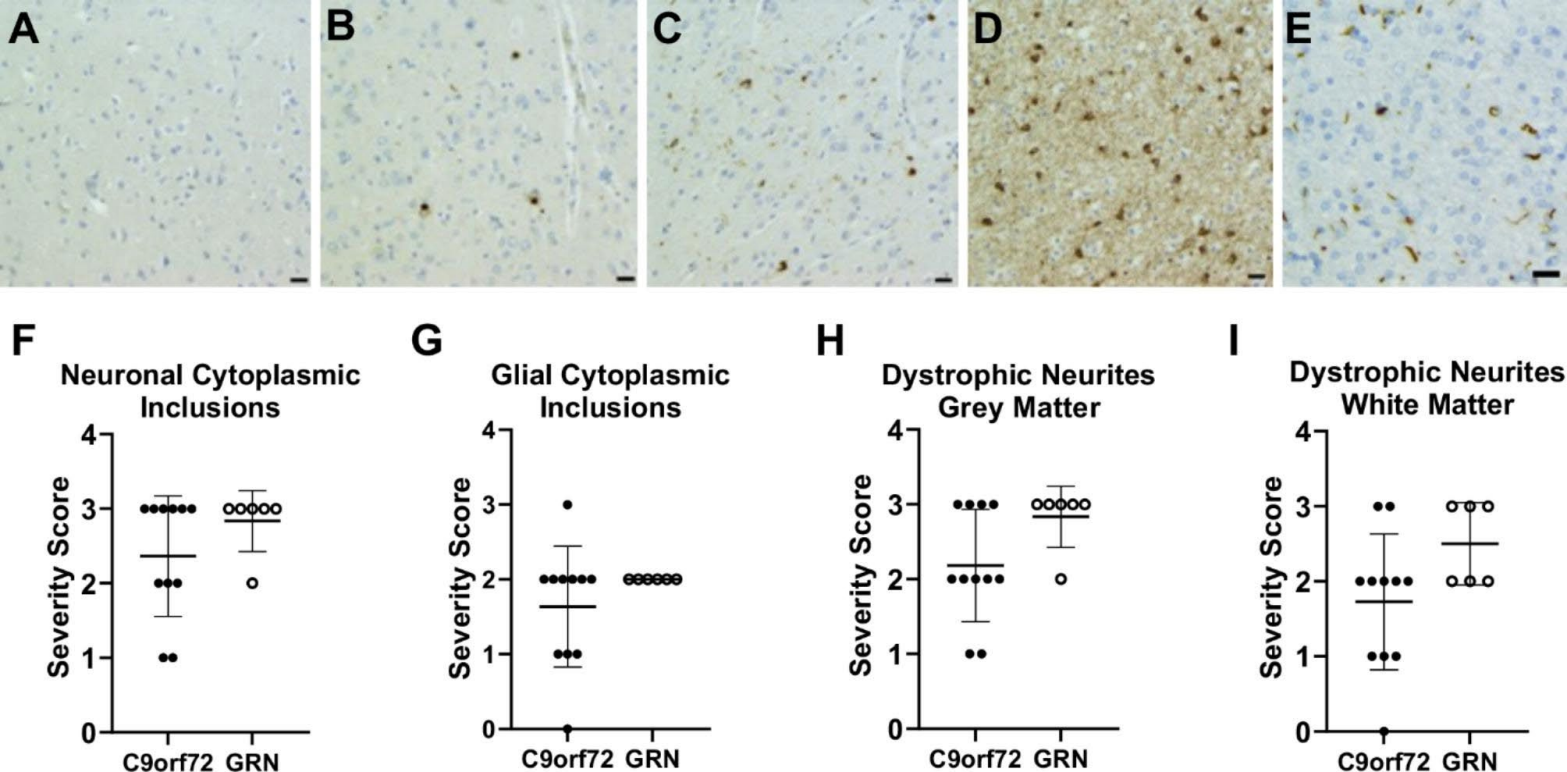
TDP-43 cytoplasmic inclusions and dystrophic neurites are similar between FTD-*GRN* and FTD-*C9orf72* cases. (A-D) Representative images of phosphorylated TDP-43 staining in grey matter, showing (A) a control case with no TDP-43 inclusions, (B) mild (score = 1), (C) moderate (score = 2), and (D) severe (score = 3) TDP-43 pathology. (E) Abundant TDP-43-positive dystrophic neurites (score = 3) in frontal white matter of an FTD case. Scale bar, 20 µM. (F-I) Severity of (F) neuronal cytoplasmic TDP-43 inclusions in frontal grey matter, (G) glial cytoplasmic inclusions in frontal white matter, (H) TDP-43-positive dystrophic neurites in frontal grey matter, and (I) dystrophic neurites in frontal white matter of FTD-*C9orf72* (n = 11) and FTD-*GRN* (n = 6) cases

This study used post-mortem brain tissue from FTD cases with *GRN* (n = 6) and *C9orf72* (n = 11) mutations, and neurologically normal controls (n = 11) (Table 1). Mean age at death was 70.3 ± 10.0 years for controls, 68.6 ± 9.3 years for *C9orf72* cases and 63.0 ± 8.8 years for *GRN* cases (ANOVA, F = 1.17, *p =* 0.33). Mean post-mortem interval (PMI) was 28.5 ± 15.5 h for controls, 20.2 ± 12.9 h for *C9orf72* cases and 25.7 ± 12.4 h for *GRN* cases (F = 0.99, *p =* 0.38). All FTD-*GRN* and 7 out of 11 FTD-*C9orf72* cases exhibited type A TDP-43 inclusions [35], with the rest exhibiting type B. The severity of TDP-43 neuronal cytoplasmic inclusions in frontal grey matter (Fig. 1A-D) or glial cytoplasmic inclusions in frontal white matter (Fig. 1E) did not differ significantly between FTD-*GRN* and FTD-*C9orf72* cases (Fig. 1F-G), nor did the severity of TDP-43-positive dystrophic neurites (Fig. 1H-I), indicating that the FTD-*GRN* and FTD-*C9orf72* cases are well-matched neuropathologically.

### Lipidomic profiles distinguishes FTD-*GRN* from control and FTD-*C9orf72* cases

**Fig. 2:**
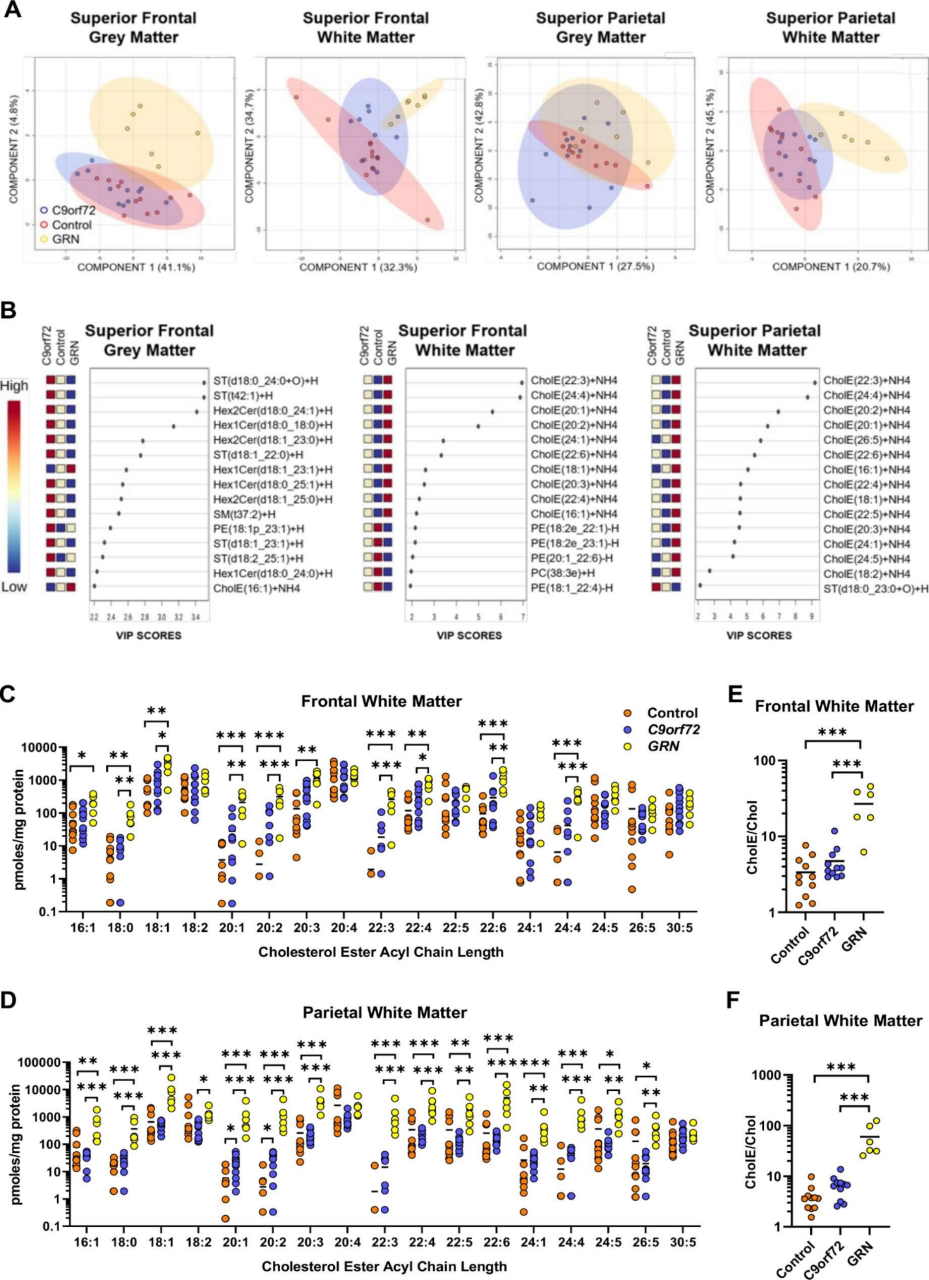
Lipid profiles distinguish FTD-*GRN* from FTD-*C9orf72* and control cases. (A) PLS-DA scores plots for control (n = 11, orange), FTD-*C9orf72* (n = 11, blue), and FTD-*GRN* (n = 6, yellow) cases for each brain region, based on the untargeted lipidomic data. Component % refers to the percentage of variance explained by each principal component. (B) Variable Importance in the Projection (VIP) scores for the 15 features that contribute most to separation of the groups in PLS-DA. ST: sulfatide, Hex1Cer: monohexosylceramide, Hex2Cer: dihexosylceramide, SM: sphingomyelin, CholE: cholesterol ester, PC: phosphatidylcholine, PE: phosphatidylethanolamine. (C,D) Levels of CholE species in (C) frontal and (D) parietal white matter. Horizontal bars show mean. Missing values indicate that the specific CholE was below the limit of detection in that sample. Sample groups were compared by one-way ANOVA adjusted for PMI and age, and *p* values were corrected for false discovery rate. Results of Tukey’s post-test are shown: *p < 0.05; **p < 0.01; ***p < 0.001. (E,F) Total cholesterol esters (CholE)/cholesterol (Chol) in (E) frontal and (F) parietal white matter

Lipidomic analysis was performed on the superior frontal grey and white matter, which are heavily affected in bvFTD, and the less affected superior parietal grey and white matter [36, 37]. A total of 821 glycerophospholipid, phospholipid, lysophospholipid, sphingolipid, sterol and acylcarnitine species were quantified (Supplementary Data File 1). Applying partial least squares discriminant analysis (PLS-DA), FTD-*GRN* cases clustered distinctly from both control and FTD-*C9orf72* cases based on lipidomic data from frontal grey matter (Fig. 2A). This was driven by myelin-enriched sphingolipids, specifically sulfatides (ST), monohexosylceramides (Hex1Cer), and dihexosylceramides (Hex2Cer) (Fig. 2B). Hex1Cer comprises both glucosylceramide and galactosylceramide, structural isomers that are indistinguishable using LC-MS/MS with reverse-phase chromatography. However, over 99% of Hex1Cer in the brain is galactosylceramide [38], which makes up 20–25% of myelin lipid [30]. ST (galactosylceramide sulfate) makes up a further 4–5% of myelin lipid [30]. PLS-DA did not effectively differentiate the three sample groups based on parietal grey matter lipids.

FTD-*GRN* cases also clustered distinctly from controls in frontal and parietal white matter (Fig. 2A), and this was driven by cholesterol esters (Fig. 2B). Many cholesterol ester species were increased by 1–2 orders of magnitude in FTD-*GRN* compared to FTD-*C9orf72* and control cases, particularly in parietal white matter (Fig. 2C-D).

### Loss of myelin lipids in FTD-*GRN* and FTD-*C9orf72* cases

**Fig. 3:**
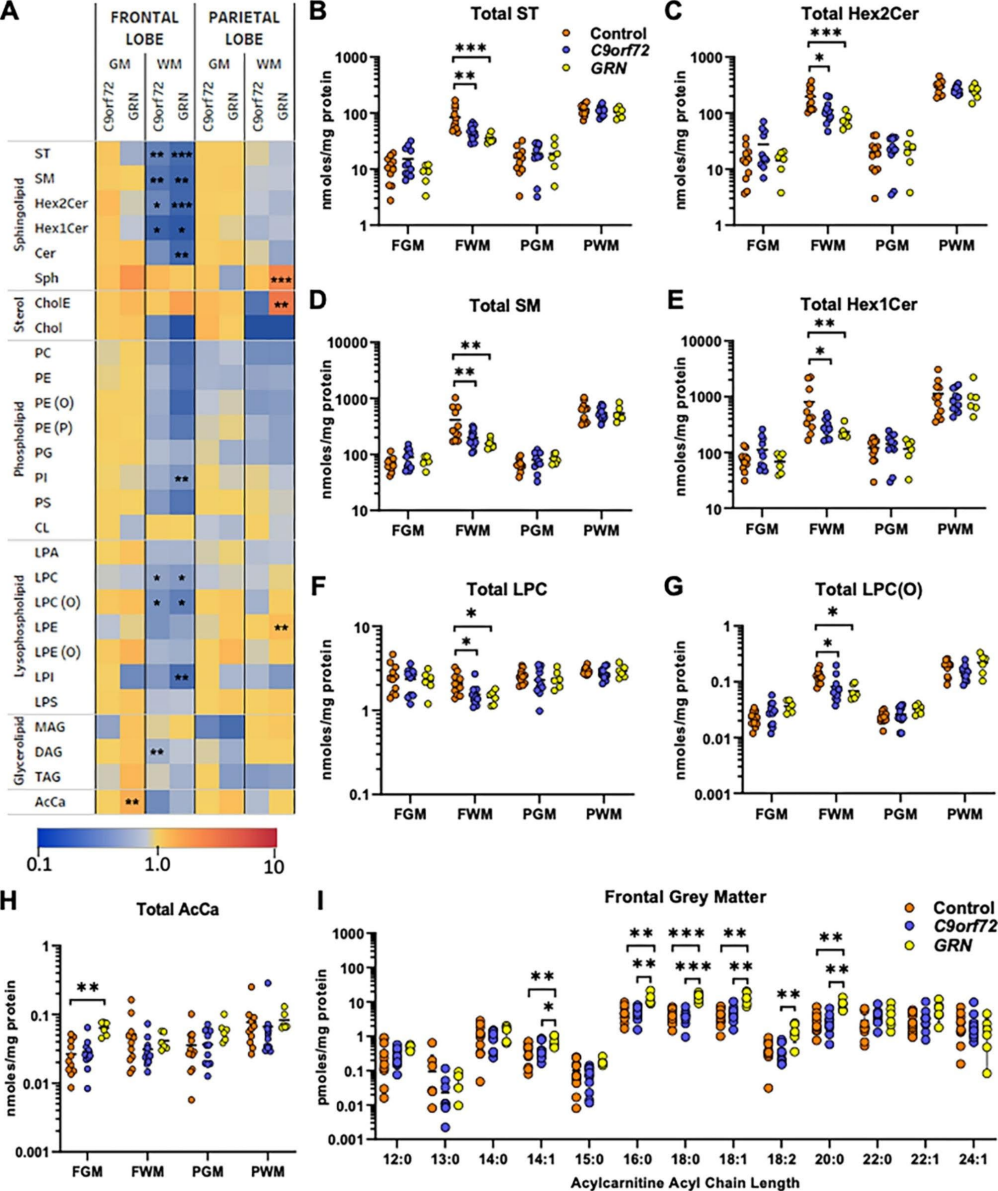
Loss of myelin lipids is common to FTD-*GRN* and FTD-*C9orf72*. (A) Mean lipid class totals in FTD-*C9orf72* (n = 11) and FTD-*GRN* (n = 6) cases, expressed as a fold-change relative to the mean of the control group (n = 11) within each brain region. GM: grey matter, WM: white matter. Sample groups were compared by one-way ANOVA adjusted for PMI and age, with *p* values adjusted for false discovery rate. Asterisks indicate a significant difference compared to the control group in Tukey’s post-test: *p < 0.05; **p < 0.01; ***p < 0.001. (B-H) Total lipid levels in each brain region. (I) Individual acylcarnitine (AcCa) species in frontal grey matter. Horizontal bar shows mean. FGM: Frontal Grey Matter; FWM: Frontal White Matter; PGM: Parietal Grey Matter; PWM: Parietal White Matter; ST: sulfatide; SM: sphingomyelin; Hex2Cer: dihexosylceramide; Hex1Cer: monohexosylceramide; Cer: ceramide; Sph: sphingosine; CholE: cholesterol ester; Chol: cholesterol; PC: phosphatidylcholine; PE: phosphatidylethanolamine; PE(O): alkyl-PE; PE(P): alkenyl-PE (PE plasmalogen); PG: phosphatidylglycerol; PI: phosphatidylinositol; PS: phosphatidylserine; CL: cardiolipin; LPA: lysophosphatidic acid; LPC: lysophosphatidylcholine; LPC(O): alkyl-LPC; LPE: lysophosphatidylethanolamine; LPE(O): alkyl-lysophosphatidylethanolamine; LPI: lysophosphatidylinositol; LPS: lysophosphatidylserine; MAG: monoacylglycerol; DAG: diacylglyerol; TAG: triacylglycerol; AcCa: acylcarnitine

To complement the unsupervised statistical analysis of individual lipids and identify metabolic nodes affected by *GRN* or C*9orf72* mutations, lipid class totals were compared across the three sample groups using ANOVA adjusted for age and PMI (Fig. 3A and Supplementary Tables 1–4). The most significant changes were observed in frontal white matter, where 9 of the 27 lipid classes were significantly reduced in the FTD-*GRN* group. Statistically significant reductions in total ST, Hex2Cer, sphingomyelin (SM), Hex1Cer, lysophosphatidylcholine (LPC), and 1-O-alkyl-lysophosphatidylcholine [LPC(O)] were observed in both FTD-*GRN* and FTD-*C9orf72* relative to control cases, although the magnitude of lipid loss was greater in FTD-*GRN* cases (Fig. 3A-G). Phosphatidylinositol (PI), lysophosphatidylinositol (LPI), and ceramide were significantly reduced in frontal white matter of FTD-*GRN* but only trended lower in FTD-*C9orf72* cases (Fig. 3A). Similar trends with an overall loss of sphingolipids and phospholipids were observed in the parietal white matter, however these were not statistically significant. Instead, total cholesterol esters, lysophosphatidylethanolamine (LPE), and sphingosine, an intermediate metabolite in sphingolipid catabolism, were significantly increased in the FTD-*GRN* group (Fig. 3A).

In addition to the sphingolipids ST, Hex1Cer, and SM, myelin is highly enriched in unesterified cholesterol [30]. Although unesterified cholesterol levels trended downwards in white matter of FTD relative to the control cases, this was not statistically significant. However, the ratio of esterified to unesterified cholesterol was an order of magnitude higher in both frontal (F = 24.6, *p* < 0.0001) and parietal (F = 49.2, *p* < 0.0001) white matter of FTD-*GRN* cases (Fig. 2E-F).

A marked increase in acylcarnitines was observed in frontal grey matter of FTD-*GRN* cases (Fig. 3A and H). Examination of individual acylcarnitine species showed a preferential effect on acylcarnitines with 16–20 carbon acyl chains (Fig. 3I). Acylcarnitines also trended higher in FTD-*GRN* cases in the parietal grey matter, however no lipid classes reached statistical significance in this region.

### FTD-*GRN* is characterised by severe myelin attrition

**Fig. 4:**
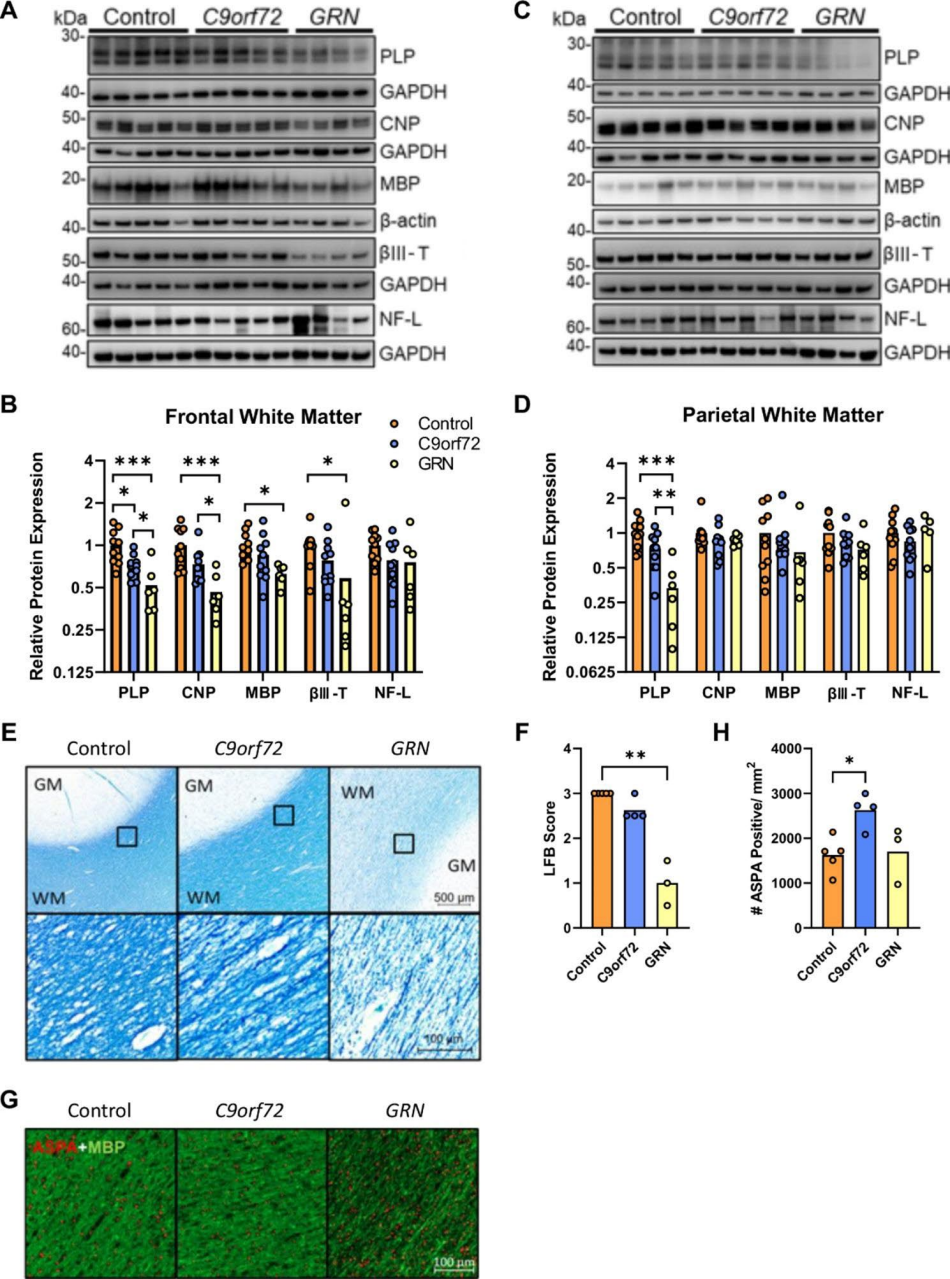
Pronounced myelin loss in FTD-*GRN*. (A, C) Representative western blots and (B, D) densitometric quantification of PLP, CNP, MBP, βIII-tubulin (βIII-T) and neurofilament light chain (NF-L) in (A, B) superior frontal white matter, and (C, D) superior parietal white matter from control (n = 11), FTD-*C9orf72* (n = 11), and FTD-*GRN* (n = 6) cases. Protein levels were normalised to β-actin or GAPDH as a loading control, and are expressed relative to the mean of the control group. (E) Representative images and (F) myelination scores from LFB staining of superior frontal gyrus white matter from control (n = 5), FTD-*C9orf72* (n = 4), and FTD-*GRN* (n = 3) cases from which tissue fixed for < 2 weeks was available. (G) Representative ASPA (red) and MBP (green) staining in superior frontal gyrus white matter, and (H) ASPA-positive cell density. Groups were compared by one-way ANOVA with Tukey’s post-test (B,D,H) or Kruskall-Wallis test with Dunn’s post-test (F): *p < 0.05; **p < 0.01; ***p < 0.001

In agreement with our lipidomic data, western blotting showed significant loss of the myelin markers proteolipid protein (PLP) (ANOVA, F = 12.03, *p =* 0.0003), 2’,3’-Cyclic nucleotide 3’-phosphodiesterase (CNP) (F = 11.64, *p =* 0.0003) and myelin basic protein (MBP) (F = 4.84, *p =* 0.018), in frontal white matter of FTD-*GRN* cases (Fig. 4A-B). Although PLP levels were reduced relative to controls in FTD-*C9orf72* cases, both PLP and CNP were significantly lower in FTD-*GRN* compared to FTD-*C9orf72* cases, indicating more severe myelin loss in FTD-*GRN* cases. In parietal white matter, PLP (F = 14.64, *p <* 0.0001) was significantly reduced in FTD-*GRN* compared to both control and FTD-*C9orf72* cases, whereas CNP (F = 0.88, *p =* 0.43) and MBP (F = 1.02, *p =* 0.38) were unchanged across the three sample groups (Fig. 4C-D). The neuronal marker βIII-tubulin was significantly decreased in frontal (Fig. 4A-B) but not parietal (Fig. 4C-D) white matter of FTD-*GRN* cases, and was not significantly affected in FTD-*C9orf72* cases (Kruskal-Wallis test, frontal white matter: H = 7.90, *p* = 0.019; parietal white matter: ANOVA F = 1.76, *p* = 0.19). Axonal marker neurofilament-L trended down in frontal white matter of FTD-*GRN* and FTD-*C9orf72* cases, however this was not statistically significant (ANOVA F = 2.32, *p* = 0.12). No difference was observed for neurofilament-L levels in parietal white matter (ANOVA F = 0.73, *p* = 0.49).

Luxol fast blue staining confirmed the pronounced myelin loss in frontal white matter of FTD-*GRN* cases (Kruskal-Wallis H = 9.47, *p =* 0.0002) (Fig. 4E-F). Loss of myelin staining in FTD-*GRN* cases was uniform and without evidence of focal lesions or plaques. Despite the pronounced myelin loss, mature oligodendrocyte (ASPA-positive) cell density [33, 39] was not reduced in frontal white matter of FTD-*GRN* cases, and was 60% higher in FTD-*C9orf72* compared to control cases (F = 6.15, *p* = 0.021) (Fig. 4G-H).

### Cholesterol esters are inversely correlated with myelin proteins and lipids

Myelin loss could explain the pronounced cholesterol ester accumulation in FTD-*GRN* cases, as cholesterol released from myelin is metabolised by phagocytic cells [40, 41]. Total cholesterol esters were inversely correlated with PLP, but not MBP or CNP, in frontal and parietal white matter (Table 2). Of all the lipids measured, ST and Hex1Cer are most unique to myelin in the CNS [42]. Cholesterol esters were inversely correlated with total Hex1Cer but not ST in frontal white matter, and both Hex1Cer and ST in parietal white matter. Since not all cholesterol esters were increased in FTD-*GRN* cases, we performed the same correlation analysis with CholE(22:6), an abundant cholesterol ester that was greatly increased in FTD-*GRN* (Fig. 1C-D). CholE(22:6) was inversely correlated with all five myelin markers (PLP, MBP, CNP, ST, Hex1Cer) in frontal white matter, and PLP, ST, and Hex1Cer in parietal white matter (Table 2). These inverse correlations support the hypothesis that cholesterol esters are indicative of myelin degradation.

**Table 2:** Correlations between cholesterol esters and myelin markers in white matter. Spearman’s correlation coefficient (r) and *p* value are shown for associations between myelin markers and total cholesterol ester (CholE) or CholE(22:6) in frontal and parietal white matter. Significant correlations are in bold font

	Frontal white matter				Parietal white matter			
	Total CholE		CholE(22:6)		Total CholE		CholE(22:6)	
	r	*p*	r	*p*	r	*p*	r	*p*
PLP	**-0.70**	**8.13 × 10** ^**− 5**^	**-0.79**	**1.53 × 10** ^**− 4**^	**-0.59**	**1.57 × 10** ^**− 3**^	**-0.66**	**2.74 × 10** ^**− 4**^
MBP	-0.20	0.32	**-0.48**	**0.014**	0.12	0.55	0.12	0.55
CNP	-0.38	0.052	**-0.43**	**0.030**	-0.10	0.64	-0.25	0.23
Total ST	-0.39	0.0501	**-0.52**	**6.07 × 10** ^**− 3**^	**-0.50**	**6.85 × 10** ^**− 3**^	**-0.52**	**4.27 × 10** ^**− 3**^
Total Hex1Cer	**-0.52**	**7.07 × 10** ^**− 3**^	**-0.46**	**0.020**	**-0.56**	**1.76 × 10** ^**− 3**^	**-0.51**	**5.73 × 10** ^**− 3**^

### Lysosomal and phagocytic markers are increased in both FTD-*GRN* and FTD-*C9orf72*

**Fig. 5:**
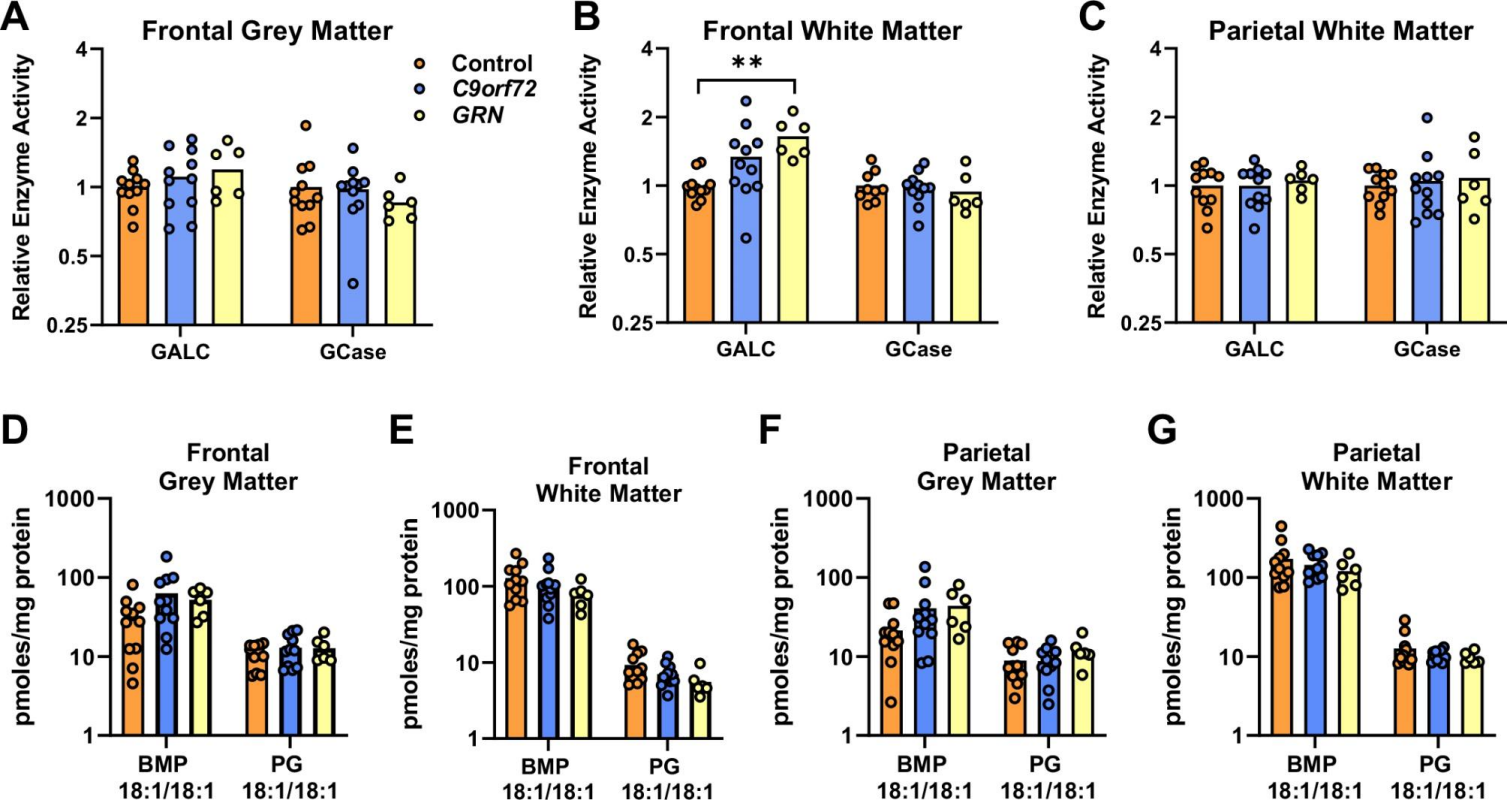
Increased GALC activity in frontal white matter of FTD-*GRN* cases. (A-C) GALC and GCase enzyme activity in frontal white matter (A), parietal white matter (B) and frontal grey matter (C) of control (n = 11), FTD-*C9orf72* (n = 11), and FTD-*GRN* (n = 6) cases. Data is normalised to the mean of the control group. (D-G) Targeted lipidomic analysis of 18:1/18:1 BMP and 18:1/18:1 PG in frontal grey matter (D), frontal white matter (E), parietal grey matter (F), parietal white matter (G). Groups were compared by one-way ANOVA with Tukey’s post-test: **p < 0.01

*GRN* mutations are proposed to disrupt lysosomal homeostasis, and hypomyelination is common to many lysosomal storage diseases, including neuronal ceroid lipofuscinosis (NCL) caused by homozygous *GRN* mutations [11, 12]. Reduced GCase activity has been reported in G*rn-/-* mice [19, 43], IPSC-derived neurons [18] and brain tissue from FTD-*GRN* cases [44, 45]. We observed no significant difference in GCase activity between FTD cases and controls, in frontal grey or white matter, or parietal white matter (Fig. 5A-C). However, galactocerebrosidase (GALC) activity, which is required for lysosomal degradation of Hex1Cer and ST, was 64% higher in frontal white matter of FTD-*GRN* (*p* = 0.004), and 33% higher in FTD-*C9orf72* cases (*p* = 0.21, not significant), relative to the controls (ANOVA F = 6.94, *p =* 0.004) (Fig. 5B).

Recent studies have also demonstrated loss of the endolysosomal lipid Bis(monoacylglycero)phosphate (BMP), particularly BMP(18:1/18:1) and BMP(22:6/22:6), in mouse models of *Grn* deficiency [19, 45]. We were able to confidently resolve the abundant BMP(18:1/18:1) species from its mass isomer PG(18:1/18:1), and show here that levels of this BMP were not significantly different in FTD cases compared to controls (Fig. 5D-G, all *p* > 0.05 by one-way ANOVA), in agreement with another recent publication [45].

**Fig. 6:**
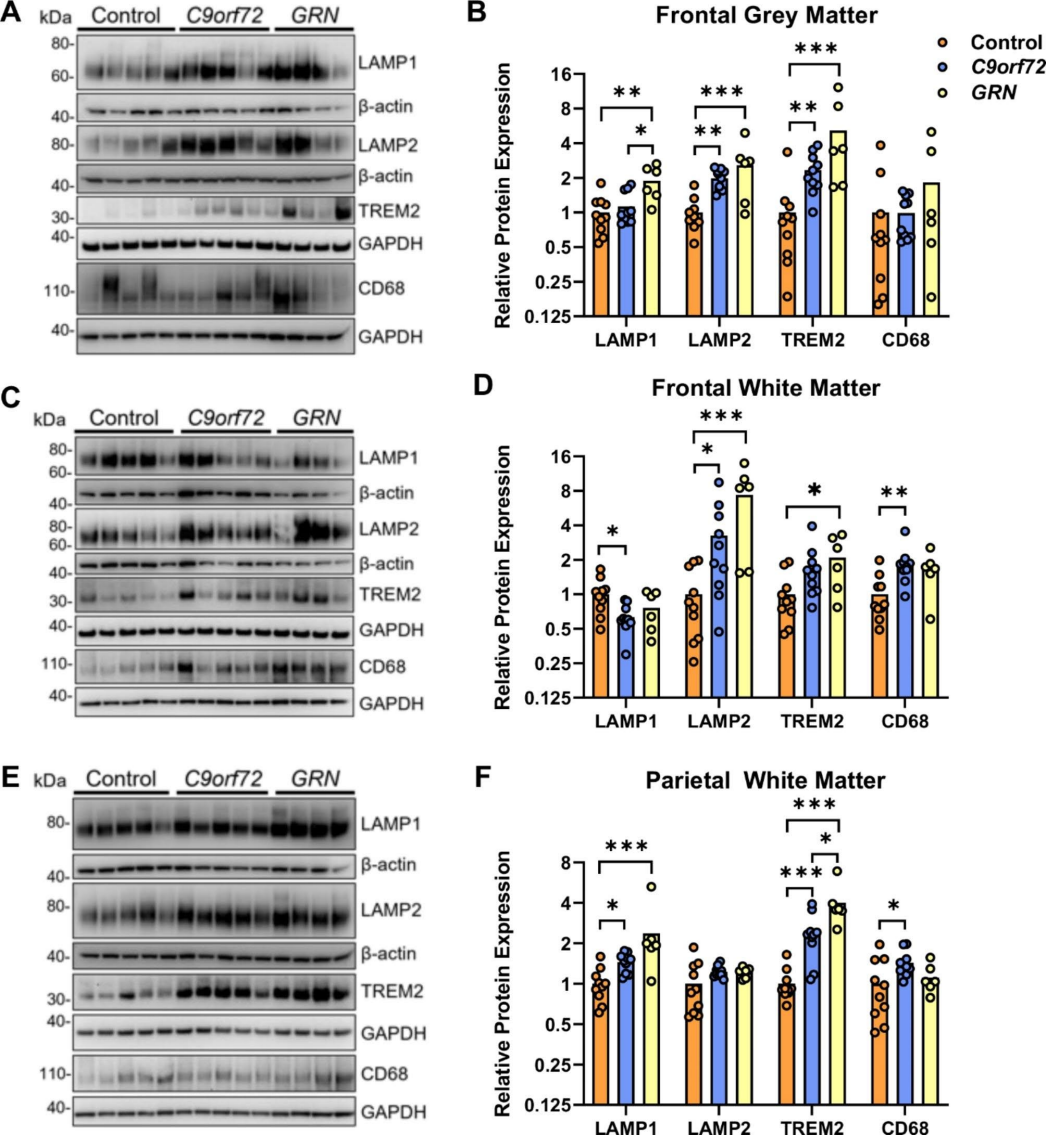
Lysosomal and phagocytic markers are increased in both FTD-*GRN* and FTD-*C9orf72*. (A, C, E) Representative western blots and (B, D, F) densitometric quantification for LAMP1, LAMP2, TREM2 and CD68 in (A, B) frontal grey matter, (C, D) frontal white matter and (E, F) parietal white matter of control (n = 11), FTD-*C9orf72* (n = 11), and FTD-*GRN* (n = 6) cases. Protein levels were normalised to β-actin or GAPDH as a loading control, and expressed relative to the mean of the control group. Asterisks indicate significant difference in Tukey’s post-test after one-way ANOVA: *p < 0.05; **p < 0.01; ***p < 0.001

Further evidence for disrupted lysosomal homeostasis in FTD-*GRN* cases has come from reports showing increased levels of lysosomal proteins such as LAMP-1 and LAMP-2 [13, 44]. Western blotting showed increased LAMP-2 levels in frontal grey (F = 12.2, *p =* 0.0003) (Fig. 6A-B) and white matter (F = 9.7, *p =* 0.0009) (Fig. 6C-D) of both FTD-*GRN* and FTD-*C9orf72* cases, whereas levels were unchanged in parietal white matter (Fig. 6E-F). LAMP-1 was increased in frontal grey matter of FTD-*GRN* cases (F = 7.0, *p =* 0.0042), and parietal white matter of both FTD-*GRN* and FTD-*C9orf72* cases (F = 11.3, *p* = 0.0004), but was reduced in the frontal white matter of FTD-*C9orf72* cases compared to controls (F = 3.9, *p =* 0.035). Levels of the phagocytic microglial marker CD68 were significantly increased in frontal (F = 6.45, *p =* 0.0049) and parietal white matter of FTD-*C9orf72* (F = 3.9, *p =* 0.034), but not FTD-*GRN* cases. However, the microglial lipid receptor TREM2 [40, 46] was increased in frontal grey matter (F = 12.5, *p =* 0.0002) and parietal white matter (F = 33.6, *p <* 0.0001) of both FTD-*GRN* and FTD-*C9orf72* cases, and frontal white matter of FTD-*GRN* cases (F = 4.7, *p =* 0.019). These results indicate that lysosomal and microglial homeostasis is disrupted in both FTD-*C9orf72* and FTD-*GRN* cases, whereas lipid and myelin protein changes are more pronounced in FTD-*GRN* cases.

## Discussion

This study presents the first comprehensive biochemical evidence that FTD-*GRN* is characterised by pronounced myelin loss. Significant loss of myelin-enriched sphingolipids was observed in white matter of both FTD-*GRN* and FTD-*C9orf72* cases, however FTD-*GRN* cases displayed a distinct lipidomic profile characterised by greater white matter lipid loss, increased levels of cholesterol esters in white matter, and increased acylcarnitines in grey matter. Levels of lysosomal markers and the microglial lipid receptor TREM2 were increased in both FTD-*C9orf72* and FTD-*GRN* cases, whereas galactocerebrosidase activity, required for catabolism of the myelin lipids galactosylceramide and ST, was only significantly increased in FTD-*GRN* cases. These changes point to a specific effect of *GRN* mutations in promoting myelin lipid catabolism and myelin degeneration, supported by the marked loss of myelin proteins and luxol fast blue staining in FTD-*GRN* relative to FTD-*C9orf72* and control cases. TDP-43 pathology did not differ significantly between the FTD-*GRN* and FTD-*C9orf72* cases, suggesting that heterozygous *GRN* mutations and *C9orf72* repeat expansions may promote TDP-43 deposition and bvFTD through distinct biochemical mechanisms.

Our study establishes that substantial white matter lipid loss is common to both FTD-*C9orf72* and FTD-*GRN*. Relative to age-matched controls, mean levels of the myelin-enriched sphingolipids ST, Hex1Cer, Hex2Cer, and SM were 43–64% lower in frontal white matter of FTD-*C9orf72* and 58–71% lower in FTD-*GRN* cases. Loss of phospholipids (PI) and lysophospholipids (LPC, LPI) in white matter of FTD-*GRN* cases probably also reflects myelin loss [42]. A previous study showed marked demyelination and gliosis in regions corresponding to white matter hyperintensities in a single FTD-*GRN* case [47]. Our study expands substantially on this, presenting several lines of biochemical evidence (myelin lipid loss, myelin protein loss, and histological staining) from multiple FTD-*GRN* cases to demonstrate that pronounced myelin loss is characteristic of FTD-*GRN*. The observation of severe myelin loss in FTD-*GRN* but not FTD-*C9orf72* cases is in agreement with reported observations of white matter hyperintensities in FTD-*GRN* but not FTD-*C9orf72* cases [25–27]. However, our demonstration of significant lipid loss and a reduction in the major myelin protein PLP in frontal white matter of FTD-*C9orf72* cases demonstrates some myelin loss, in agreement with a recent study reporting decreased MBP immunoreactivity in frontal cortex of FTD-*C9orf72* cases [48]. Mature oligodendrocyte density did not decrease in FTD-*GRN* cases, implying that demyelination does not stem from oligodendrocyte loss. However, given the significant frontal lobe atrophy in bvFTD [1], equivalent oligodendrocyte density likely indicates an overall loss of oligodendrocytes relative to the age-matched controls. This could explain the increased mature oligodendrocyte density in *FTD-C9orf72* cases.

FTD-*GRN* were differentiated from FTD-*C9orf72* cases by pronounced accumulation of cholesterol esters in white matter, relative to both protein content and unesterified cholesterol. Boland et al. very recently reported a modest increase in sterol esters in middle frontal gyrus of FTD cases with *GRN* mutations but not sporadic cases with TPD-43 inclusions [45], however it was unclear if grey or white matter was used and control cases were limiting. We observed significantly-increased cholesterol esters only in white matter. Cholesterol esters are formed during myelin break-down and accumulate in demyelinating conditions, as microglia and infiltrating macrophages phagocytose cholesterol released from compact myelin [40, 41, 49, 50]. Since cholesterol cannot be broken down by CNS cells, this free cholesterol is stored in esterified form and eventually cleared by excretion [51]. In the CNS, myelin phagocytosis and degradation is carried out by microglia and macrophages [40, 41, 52], which are the cell types that express *GRN* most abundantly. Although cholesterol ester accumulation in white matter of FTD-*GRN* cases is probably associated with excessive myelin break-down, it is also possible that this phenotype results from a defect in cholesterol break-down and clearance caused by *GRN* haploinsufficiency. In this regard, a recent study reported that *GRN* deficiency impairs clearance of myelin debris by cultured microglia [53]. Cholesterol overload in microglia triggers lysosome rupture and NLRP3 inflammasome activation [41], which could fuel neuroinflammation in FTD-*GRN* cases. Cholesterol ester formation also appears to be an important driver of amyloid β and neurofibrillary tangle pathology in Alzheimer’s disease models [54, 55].

Diffusion tensor imaging studies have shown loss of white matter integrity in *C9orf72*, *GRN* and *MAPT* mutation carriers up to 30 years prior to estimated symptom onset [28, 29], suggesting that myelin deterioration begins early in FTD pathogenesis. Using post-mortem tissue samples, it is difficult to determine if white matter changes precede axon degeneration, or vice-versa. Loss of myelin markers in frontal white matter of FTD-*GRN* cases was accompanied by a significant reduction in βIII-tubulin but not neurofilament-L by western blotting. In contrast, we observed significant myelin lipid loss in FTD-*C9orf72* cases without evidence for loss of axonal markers by western blotting, although noting that this could be attributed to the more quantitative nature of our lipidomic analysis in comparison to western blotting and densitometry. In the less affected parietal white matter, the pronounced increase in cholesterol esters without significant loss of myelin lipids or axonal markers suggests that cholesterol ester storage is an early phenotype resulting from *GRN* haploinsufficiency.

Another differentiating feature of FTD-*GRN* was increased levels of long chain (C16-C20) acylcarnitines in frontal grey matter. Acylcarnitines are formed to import fatty acids into mitochondria for β-oxidation, and their accumulation is commonly associated with impaired β-oxidation of fatty acids [56]. Defective fatty acid β-oxidation can produce a brain energy deficit, which could partly explain the hypometabolic phenotype of FTD [57]. Acylcarnitine accumulation attributed to impaired β-oxidation of very long chain fatty acids in peroxisomes is a defining feature of X-linked adrenoleukodystrophy [58], also characterised by cholesterol ester accumulation [50]. Further research is required to determine if impaired fatty acid oxidation is a feature of FTD-*GRN*. Alternatively, the increased acylcarnitines could be indicative of a metabolic shift favouring lipid oxidation for energy production at the expense of lipid synthesis, thus causing myelin degeneration [59].

Levels of the microglial lipid receptor TREM2 were significantly higher in grey and white matter of both FTD-*GRN* and FTD-*C9orf72* cases, indicating the presence of phagocytic microglia. Lipid sensing by TREM2 promotes microglial activation and myelin phagocytosis [40, 52]. Our data therefore provides important evidence from human FTD cases confirming the observation that microglia from *C9orf72* and *Grn* knockout mice exhibit a phagocytic microglial phenotype [60, 61]. In addition to phagocytosing myelin, activated microglia promote neurodegeneration through increased secretion of inflammatory cytokines and complement proteins, and synaptic pruning [61, 62]. In fact, complement proteins secreted by *GRN*-/- microglia are sufficient to induce TDP-43 granules and cell death in excitatory neurons [62]. Our data also establishes that lysosomal protein markers LAMP-1, LAMP-2, and CD68 are deregulated in FTD-*C9orf72* cases, and demonstrates that this phenotype is shared with FTD-*GRN*. C9orf72 colocalises with Rab family proteins and regulates endocytosis, lysosome biogenesis and phagosome maturation [23, 63]. Prior work had shown increased LAMP-1 and CD68 immunoreactivity in ALS cases with *C9orf72* repeat expansions [63, 64], however *C9orf72-/-* motor neurons and those from ALS-*C9orf72* cases have fewer lysosomes, despite higher LAMP-2 content in lysosomal membranes [23].

Current evidence suggests that progranulin is required for full activity of lysosomal lipid hydrolases, particularly GCase [19, 43, 44]. Higher levels of the GCase substrate glucosylsphingosine have been reported in plasma of FTD-*GRN* cases [19], whereas another study showed unchanged levels in the inferior frontal gyrus [44]. Decreased GCase activity has been attributed to a role for progranulin in regulating the delivery of prosaposin to lysosomes and its proteolytic cleavage into saposins [10, 18], which serve as cofactors for lysosomal sphingolipid hydrolases such as GCase. Others have proposed a direct interaction between progranulin and GCase [43], or that progranulin regulates lysosomal enzyme functions through a direct interaction with BMP [19, 45]. Further evidence for lysosomal dysfunction comes from the recent demonstration of gangliosidosis in frontal cortex of FTD-*GRN* cases [45]. We did not find reduced BMP content in FTD-*GRN* cases compared to controls, indicating that not all molecular phenotypes of *Grn-/-* mice accurately reflect changes in FTD cases with heterozygous *GRN* mutations. GCase activity trended lower (14% reduction) in frontal grey matter of FTD-*GRN* cases, in line with modest reductions reported in two prior studies [44, 45], however this was not statistically significant. We note that GCase activity reductions are modest even in *Grn-/-* mice, with one study reporting a decrease of ~ 10% [44], and were not seen in mice bearing the R493X *Grn* mutation found in FTD [45].

In contrast to the absence of any change in GCase activity, the clear and significant increase in GALC activity in frontal white matter of FTD-*GRN* cases aligns with our observation of significantly reduced sulfatide and Hex1Cer levels in the same samples. GALC activity in frontal white matter of FTD-*C9orf72* cases was lower than FTD-*GRN* but higher than the control cases (not significant), in agreement with the lipidomic results. Increased GALC (b-galactosidase) activity was also seen in frontal cortex of *Grn*-/- mice [44], and *GALC* RNA levels are higher in motor cortex of ALS cases compared to age-matched controls [65]. Overall, decreased sphingolipids in FTD-*GRN* cases, together with increased levels of the sphingolipid catabolic intermediate sphingosine in parietal white matter, support the concept that *GRN* mutations disrupt brain sphingolipid metabolism. Future studies with cell culture models will be necessary to resolve whether *GRN* haploinsufficiency causes a block in lysosomal catabolism that leads to accumulation of cholesterol esters, sphingosine, and acylcarnitines; or whether these features are biomarkers of accelerated myelin break-down, as indicated by the increased GALC activity in frontal white matter.

Our lipidomic results with FTD cases are in broad agreement with a recent paper reporting decreased SM, ceramide, and some phospholipids, and increased cholesterol esters and triglycerides, in motor cortex white matter of ALS cases [65]. Decreased myelin-enriched sphingolipids (GalCer, ST, SM) are also observed in motor cortex of people with multiple system atrophy [66], which is characterised by a-synuclein aggregates in oligodendrocytes. In contrast, the more common synucleinopathy Parkinson’s disease is characterised by increased BMP in the heavily-affected substantia nigra [67], and increased diacylglycerol in frontal cortex [68]. In Alzheimer’s disease (AD), marked depletion of myelin sphingolipids and myelin proteins was seen in superior frontal grey matter, but not frontal white matter [69, 70]. Given that both AD and bvFTD affect the superior frontal lobe, it is interesting that the pattern of myelin loss differs between AD and bvFTD, with a much more pronounced effect on the frontal white matter seen in the familial bvFTD cases examined herein. Accordingly, the burden of white matter hyperintensities is higher in bvFTD than AD and is thought to contribute substantially to cognitive deficits [71, 72].

A limitation of our study was the absence of sporadic FTD cases. It will be important in future studies to determine whether the lipidomic signature and pronounced myelin loss in FTD-*GRN* cases is shared with a subset of sporadic FTD cases with TDP-43 inclusions. This seems likely, since (i) MRI studies have demonstrated loss of white matter integrity in sporadic bvFTD [72, 73] and (ii) rare variants in genes whose loss of function is associated with the severe inherited leukodystrophies hypomyelinating leukodystrophy (*TMEM106B* gene), Nasu-Hakola disease (*TREM2*), metachromatic leukodystrophy (*ARSA*), and cerebrotendinous xanthomatosis (*CYP27A1*), are also known to cause FTD with TDP-43 deposition [74].

In conclusion, this study presents the first evidence of severe myelin lipid loss in FTD-*GRN* and FTD-*C9orf72*. More severe white matter lipid and myelin protein loss in FTD-*GRN*, together with marked accumulation of cholesterol esters in white matter and increased GALC activity, imply a pronounced susceptibility for myelin lipid loss, leading to white matter attrition, in *GRN* mutation carriers. These results are consistent with a requirement for progranulin in restricting myelin lipid catabolism. In fact, our data shows that FTD-*GRN* displays features of metabolic leukodystrophies, including myelin loss, gliosis, and cholesterol ester storage [50]. MRI studies underscore the importance of myelin attrition in the behavioural deficits that define bvFTD [71, 72], and our data provides biochemical evidence underpinning the use of myelin MRI as a diagnostic and prognostic tool in FTD management. Since myelin is essential for neuronal health and neurological functions, accelerated myelin loss may be a key driver of neurodegeneration caused by progranulin haploinsufficiency.

## Electronic supplementary material

**Supplementary Data File 1**

**Supplementary Table 1**. Lipid Class Totals (nmoles/mg protein) in frontal white matter and one-way ANOVA results. BH FDR: Benjamini-Hochberg false discovery rate-corrected p value (q value). P values for Tukey’s post-test are also given. **Supplementary Table 2**. Lipid Class Totals (nmoles/mg protein) in frontal grey matter and one-way ANOVA results. BH FDR: Benjamini-Hochberg false discovery rate-corrected p value (q value). P values for Tukey’s post-test are also given. **Supplementary Table 3**. Lipid Class Totals (nmoles/mg protein) in parietal white matter and one-way ANOVA results. BH FDR: Benjamini-Hochberg false discovery rate-corrected p value (q value). P values for Tukey’s post-test are also given. **Supplementary Table 4**. Lipid Class Totals (nmoles/mg protein) in parietal grey matter and one-way ANOVA results. BH FDR: Benjamini-Hochberg false discovery rate-corrected p value (q value). P values for Tukey’s post-test are also given.

## Data Availability

The complete lipidomic dataset is included as Supplementary Data File 1 and available at Metabolomics Workbench [75], study ST002452, DOI: 10.21228/M8BD85. All other raw data is available from the corresponding author upon reasonable request.

## List of Abbreviations

AcCa: Acylcarnitine
AD: Alzheimer’s disease
ALS: Amyotrophic Lateral Sclerosis
ASPA: Aspartoacylase
BMP: Bis(Monoacylglycero)Phosphate
BSA: Bovine Serum Albumin
bvFTD: Behavioural Variant FTD
C9orf72: Chromosome 9 open reading frame 72
Cer: Ceramide
Chol: Cholesterol
CholE: Cholesterol Ester
CL: Cardiolipin
CNP: 2’,3’-Cyclic Nucleotide 3’-Phosphodiesterase
DAG: Diacylglyerol
DAPI: Diamidino-2-Phenylindole Dihydrochloride
FTD: Frontotemporal Dementia
GALC: β-Galactocerebrosidase
GCase: β-Glucocerebrosidase
GRN: Progranulin
Hex1Cer: Monohexosylceramide
Hex2Cer: Dihexosylceramide
LFB: Luxol Fast Blue
LPA: Lysophosphatidic acid
LPC: Lysophosphatidylcholine
LPC(O): Alkyl-LPC
LPE: Lysophosphatidylethanolamine
LPE(O): Alkyl-Lysophosphatidylethanolamine
LPI: Lysophosphatidylinositol
LPS: Lysophosphatidylserine
MAG: Monoacylglycerol
MBP: Myelin Basic Protein
MRI: Magnetic Resonance Imaging
MTBE: Methyl-Tert-Butyl Ether
NCL: Neuronal Ceroid Lipofuscinosis
PC: Phosphatidylcholine
PE: Phosphatidylethanolamine
PE(O): Alkyl-PE
PE(P): Alkenyl-PE(PE plasmalogen)
PG: Phosphatidylglycerol
PI: Phosphatidylinositol
PLP: Myelin Proteolipid Protein
PLS-DA: Partial Least Squares-Discriminant Analysis
PMI: Post-Mortem Interval
PS: Phosphatidylserine
SM: Sphingomyelin
Sph: Sphingosine
ST: Sulfatide
TAG: Triacylglycerol
TBST: Tris Buffered Saline, 0.1% Tween 20
TDP-43: TAR DNA-binding protein 43

## References

[1] Olney NT, Spina S, Miller BL (2017) Frontotemporal Dementia. Neurologic clinics 35:339 – 74. 10.1016/j.ncl.2017.01.008 10.1016/j.ncl.2017.01.008 PMC5472209 28410663

[2] Hodges JR, Davies R, Xuereb J, Kril J, Halliday G (2003). Survival in frontotemporal dementia. Neurology.

[3] Greaves CV, Rohrer JD (2019). An update on genetic frontotemporal dementia. J Neurol.

[4] Neumann M, Sampathu DM, Kwong LK, Truax AC, Micsenyi MC, Chou TT, Bruce J, Schuck T, Grossman M, Clark CM, McCluskey LF, Miller BL, Masliah E, Mackenzie IR, Feldman H, Feiden W, Kretzschmar HA, Trojanowski JQ, Lee VM (2006). Ubiquitinated TDP-43 in frontotemporal lobar degeneration and amyotrophic lateral sclerosis. Science.

[5] Cruts M, Gijselinck I, van der Zee J, Engelborghs S, Wils H, Pirici D, Rademakers R, Vandenberghe R, Dermaut B, Martin JJ, van Duijn C, Peeters K, Sciot R, Santens P, De Pooter T, Mattheijssens M, Van den Broeck M, Cuijt I, Vennekens K, De Deyn PP, Kumar-Singh S, Van Broeckhoven C (2006). Null mutations in progranulin cause ubiquitin-positive frontotemporal dementia linked to chromosome 17q21. Nature.

[6] Baker M, Mackenzie IR, Pickering-Brown SM, Gass J, Rademakers R, Lindholm C, Snowden J, Adamson J, Sadovnick AD, Rollinson S, Cannon A, Dwosh E, Neary D, Melquist S, Richardson A, Dickson D, Berger Z, Eriksen J, Robinson T, Zehr C, Dickey CA, Crook R, McGowan E, Mann D, Boeve B, Feldman H, Hutton M (2006). Mutations in progranulin cause tau-negative frontotemporal dementia linked to chromosome 17. Nature.

[7] Majounie E, Renton AE, Mok K, Dopper EGP, Waite A, Rollinson S, Chiò A, Restagno G, Nicolaou N, Simon-Sanchez J, van Swieten JC, Abramzon Y, Johnson JO, Sendtner M, Pamphlett R, Orrell RW, Mead S, Sidle KC, Houlden H, Rohrer JD, Morrison KE, Pall H, Talbot K, Ansorge O, Hernandez DG, Arepalli S, Sabatelli M, Mora G, Corbo M, Giannini F, Calvo A, Englund E, Borghero G, Floris GL, Remes AM, Laaksovirta H, McCluskey L, Trojanowski JQ, Van Deerlin VM, Schellenberg GD, Nalls MA, Drory VE, Lu C-S, Yeh T-H, Ishiura H, Takahashi Y, Tsuji S, Le Ber I, Brice A, Drepper C, Williams N, Kirby J, Shaw P, Hardy J, Tienari PJ, Heutink P, Morris HR, Pickering-Brown S, Traynor BJ (2012). Frequency of the C9orf72 hexanucleotide repeat expansion in patients with amyotrophic lateral sclerosis and frontotemporal dementia: a cross-sectional study. Lancet Neurol.

[8] DeJesus-Hernandez M, Mackenzie IR, Boeve BF, Boxer AL, Baker M, Rutherford NJ, Nicholson AM, Finch NA, Flynn H, Adamson J, Kouri N, Wojtas A, Sengdy P, Hsiung G-YR, Karydas A, Seeley WW, Josephs KA, Coppola G, Geschwind DH, Wszolek ZK, Feldman H, Knopman DS, Petersen RC, Miller BL, Dickson DW, Boylan KB, Graff-Radford NR, Rademakers R (2011). Expanded GGGGCC hexanucleotide repeat in noncoding region of C9ORF72 causes chromosome 9p-linked FTD and ALS. Neuron.

[9] Hu F, Padukkavidana T, Vaegter CB, Brady OA, Zheng Y, Mackenzie IR, Feldman HH, Nykjaer A, Strittmatter SM (2010). Sortilin-mediated endocytosis determines levels of the frontotemporal dementia protein, progranulin. Neuron.

[10] Zhou X, Sun L, Bracko O, Choi JW, Jia Y, Nana AL, Brady OA, Hernandez JCC, Nishimura N, Seeley WW, Hu F (2017). Impaired prosaposin lysosomal trafficking in frontotemporal lobar degeneration due to progranulin mutations. Nat Commun.

[11] Smith KR, Damiano J, Franceschetti S, Carpenter S, Canafoglia L, Morbin M, Rossi G, Pareyson D, Mole SE, Staropoli JF, Sims KB, Lewis J, Lin WL, Dickson DW, Dahl HH, Bahlo M, Berkovic SF (2012). Strikingly different clinicopathological phenotypes determined by progranulin-mutation dosage. Am J Hum Genet.

[12] Almeida MR, Macário MC, Ramos L, Baldeiras I, Ribeiro MH, Santana I (2016) Portuguese family with the co-occurrence of frontotemporal lobar degeneration and neuronal ceroid lipofuscinosis phenotypes due to progranulin gene mutation. Neurobiology of Aging 41:200.e1-00.e5 10.1016/j.neurobiolaging.2016.02.019 27021778

[13] Gotzl JK, Mori K, Damme M, Fellerer K, Tahirovic S, Kleinberger G, Janssens J, van der Zee J, Lang CM, Kremmer E, Martin JJ, Engelborghs S, Kretzschmar HA, Arzberger T, Van Broeckhoven C, Haass C, Capell A (2014). Common pathobiochemical hallmarks of progranulin-associated frontotemporal lobar degeneration and neuronal ceroid lipofuscinosis. Acta Neuropathol.

[14] Ahmed Z, Sheng H, Xu YF, Lin WL, Innes AE, Gass J, Yu X, Wuertzer CA, Hou H, Chiba S, Yamanouchi K, Leissring M, Petrucelli L, Nishihara M, Hutton ML, McGowan E, Dickson DW, Lewis J (2010). Accelerated lipofuscinosis and ubiquitination in granulin knockout mice suggest a role for progranulin in successful aging. Am J Pathol.

[15] Ward ME, Chen R, Huang HY, Ludwig C, Telpoukhovskaia M, Taubes A, Boudin H, Minami SS, Reichert M, Albrecht P, Gelfand JM, Cruz-Herranz A, Cordano C, Alavi MV, Leslie S, Seeley WW, Miller BL, Bigio E, Mesulam MM, Bogyo MS, Mackenzie IR, Staropoli JF, Cotman SL, Huang EJ, Gan L, Green AJ (2017) Individuals with progranulin haploinsufficiency exhibit features of neuronal ceroid lipofuscinosis. Sci Transl Med 9. 10.1126/scitranslmed.aah5642 10.1126/scitranslmed.aah5642 PMC5526610 28404863

[16] Evers BM, Rodriguez-Navas C, Tesla RJ, Prange-Kiel J, Wasser CR, Yoo KS, McDonald J, Cenik B, Ravenscroft TA, Plattner F, Rademakers R, Yu G, White CL 3rd, Herz J (2017) Lipidomic and transcriptomic basis of lysosomal dysfunction in Progranulin Deficiency. Cell Rep 20:2565–2574. 10.1016/j.celrep.2017.08.056 10.1016/j.celrep.2017.08.056 PMC5757843 28903038

[17] Tanaka Y, Suzuki G, Matsuwaki T, Hosokawa M, Serrano G, Beach TG, Yamanouchi K, Hasegawa M, Nishihara M (2017). Progranulin regulates lysosomal function and biogenesis through acidification of lysosomes. Hum Mol Genet.

[18] Valdez C, Ysselstein D, Young TJ, Zheng J, Krainc D (2020). Progranulin mutations result in impaired processing of prosaposin and reduced glucocerebrosidase activity. Hum Mol Genet.

[19] Logan T, Simon MJ, Rana A, Cherf GM, Srivastava A, Davis SS, Low RLY, Chiu CL, Fang M, Huang F, Bhalla A, Llapashtica C, Prorok R, Pizzo ME, Calvert MEK, Sun EW, Hsiao-Nakamoto J, Rajendra Y, Lexa KW, Srivastava DB, van Lengerich B, Wang J, Robles-Colmenares Y, Kim DJ, Duque J, Lenser M, Earr TK, Nguyen H, Chau R, Tsogtbaatar B, Ravi R, Skuja LL, Solanoy H, Rosen HJ, Boeve BF, Boxer AL, Heuer HW, Dennis MS, Kariolis MS, Monroe KM, Przybyla L, Sanchez PE, Meisner R, Diaz D, Henne KR, Watts RJ, Henry AG, Gunasekaran K, Astarita G, Suh JH, Lewcock JW, DeVos SL, Di Paolo G (2021) Rescue of a lysosomal storage disorder caused by Grn loss of function with a brain penetrant progranulin biologic. Cell 184:4651-68 e25. 10.1016/j.cell.2021.08.002 10.1016/j.cell.2021.08.002 PMC8489356 34450028

[20] Mori K, Weng SM, Arzberger T, May S, Rentzsch K, Kremmer E, Schmid B, Kretzschmar HA, Cruts M, Van Broeckhoven C, Haass C, Edbauer D (2013) The C9orf72 GGGGCC repeat is translated into aggregating dipeptide-repeat proteins in FTLD/ALS. Science 339:1335-8. 10.1126/science.1232927 10.1126/science.1232927 23393093

[21] Gendron TF, Bieniek KF, Zhang YJ, Jansen-West K, Ash PE, Caulfield T, Daughrity L, Dunmore JH, Castanedes-Casey M, Chew J, Cosio DM, van Blitterswijk M, Lee WC, Rademakers R, Boylan KB, Dickson DW, Petrucelli L (2013). Antisense transcripts of the expanded C9ORF72 hexanucleotide repeat form nuclear RNA foci and undergo repeat-associated non-ATG translation in c9FTD/ALS. Acta Neuropathol.

[22] Kwon I, Xiang S, Kato M, Wu L, Theodoropoulos P, Wang T, Kim J, Yun J, Xie Y, McKnight SL (2014). Poly-dipeptides encoded by the C9orf72 repeats bind nucleoli, impede RNA biogenesis, and kill cells. Science.

[23] Shi Y, Lin S, Staats KA, Li Y, Chang WH, Hung ST, Hendricks E, Linares GR, Wang Y, Son EY, Wen X, Kisler K, Wilkinson B, Menendez L, Sugawara T, Woolwine P, Huang M, Cowan MJ, Ge B, Koutsodendris N, Sandor KP, Komberg J, Vangoor VR, Senthilkumar K, Hennes V, Seah C, Nelson AR, Cheng TY, Lee SJ, August PR, Chen JA, Wisniewski N, Hanson-Smith V, Belgard TG, Zhang A, Coba M, Grunseich C, Ward ME, van den Berg LH, Pasterkamp RJ, Trotti D, Zlokovic BV, Ichida JK (2018). Haploinsufficiency leads to neurodegeneration in C9ORF72 ALS/FTD human induced motor neurons. Nat Med.

[24] Farg MA, Sundaramoorthy V, Sultana JM, Yang S, Atkinson RAK, Levina V, Halloran MA, Gleeson PA, Blair IP, Soo KY, King AE, Atkin JD (2014). C9ORF72, implicated in amytrophic lateral sclerosis and frontotemporal dementia, regulates endosomal trafficking. Hum Mol Genet.

[25] Sudre CH, Bocchetta M, Cash D, Thomas DL, Woollacott I, Dick KM, van Swieten J, Borroni B, Galimberti D, Masellis M, Tartaglia MC, Rowe JB, Graff C, Tagliavini F, Frisoni G, Laforce R Jr, Finger E, de Mendonca A, Sorbi S, Ourselin S, Cardoso MJ, Rohrer JD (2017) Genetic Ftd Initiative G White matter hyperintensities are seen only in GRN mutation carriers in the GENFI cohort. Neuroimage Clin 15:171 – 80. 10.1016/j.nicl.2017.04.015 10.1016/j.nicl.2017.04.015 PMC5429247 28529873

[26] Paternico D, Premi E, Gazzina S, Cosseddu M, Alberici A, Archetti S, Cotelli MS, Micheli A, Turla M, Gasparotti R, Padovani A, Borroni B (2016). White matter hyperintensities characterize monogenic frontotemporal dementia with granulin mutations. Neurobiol Aging.

[27] Ameur F, Colliot O, Caroppo P, Stroer S, Dormont D, Brice A, Azuar C, Dubois B, Le Ber I, Bertrand A (2016). White matter lesions in FTLD: distinct phenotypes characterize GRN and C9ORF72 mutations. Neurol Genet.

[28] Jiskoot LC, Bocchetta M, Nicholas JM, Cash DM, Thomas D, Modat M, Ourselin S, Rombouts S, Dopper EGP, Meeter LH, Panman JL, van Minkelen R, van der Ende EL, Donker Kaat L, Pijnenburg YAL, Borroni B, Galimberti D, Masellis M, Tartaglia MC, Rowe J, Graff C, Tagliavini F, Frisoni GB, Laforce R Jr, Finger E, de Mendonca A, Sorbi S, Genetic Frontotemporal dementia I, Papma JM, van Swieten JC, Rohrer JD (2018) Presymptomatic white matter integrity loss in familial frontotemporal dementia in the GENFI cohort: A cross-sectional diffusion tensor imaging study. Ann Clin Transl Neurol 5:1025-36. 10.1002/acn3.601 10.1002/acn3.601 PMC6144447 30250860

[29] Mahoney CJ, Simpson IJ, Nicholas JM, Fletcher PD, Downey LE, Golden HL, Clark CN, Schmitz N, Rohrer JD, Schott JM, Zhang H, Ourselin S, Warren JD, Fox NC (2015). Longitudinal diffusion tensor imaging in frontotemporal dementia. Ann Neurol.

[30] Quarles RH, Macklin WB, Morell P (2006) Myelin formation, structure and Biochemistry. In: Brady ST et al (eds) Basic Neurochemistry: Molecular, Cellular and Medical Aspects. Elsevier, pp 51–71

[31] Couttas TA, Rustam YH, Song H, Qi Y, Teo JD, Chen J, Reid GE, Don AS (2020) A Novel Function of Sphingosine Kinase 2 in the Metabolism of Sphinga-4,14-Diene Lipids. Metabolites 10. 10.3390/metabo10060236 10.3390/metabo10060236 PMC7344861 32521763

[32] Oftedal L, Maple-Grødem J, Førland MGG, Alves G, Lange J (2020). Validation and assessment of preanalytical factors of a fluorometric in vitro assay for glucocerebrosidase activity in human cerebrospinal fluid. Sci Rep.

[33] Song H, McEwen HP, Duncan T, Lee JY, Teo JD, Don AS (2021). Sphingosine kinase 2 is essential for remyelination following cuprizone intoxication. Glia.

[34] Bankhead P, Loughrey MB, Fernández JA, Dombrowski Y, McArt DG, Dunne PD, McQuaid S, Gray RT, Murray LJ, Coleman HG, James JA, Salto-Tellez M, Hamilton PW (2017). QuPath: open source software for digital pathology image analysis. Sci Rep.

[35] Mackenzie IR, Neumann M, Baborie A, Sampathu DM, Du Plessis D, Jaros E, Perry RH, Trojanowski JQ, Mann DM, Lee VM (2011). A harmonized classification system for FTLD-TDP pathology. Acta Neuropathol.

[36] Kril JJ, Halliday GM (2004). Clinicopathological staging of frontotemporal dementia severity: correlation with regional atrophy. Dement Geriatr Cogn Disord.

[37] Manera AL, Dadar M, Collins DL, Ducharme S, Frontotemporal Lobar Degeneration Neuroimaging I (2019). Deformation based morphometry study of longitudinal MRI changes in behavioral variant frontotemporal dementia. NeuroImage Clin.

[38] Vanier MT, Svennerholm L (1975). Chemical pathology of Krabbe’s disease. III. Ceramide-hexosides and gangliosides of brain. Acta Paediatr Scand.

[39] Madhavarao CN, Moffett JR, Moore RA, Viola RE, Namboodiri MA, Jacobowitz DM (2004). Immunohistochemical localization of aspartoacylase in the rat central nervous system. J Comp Neurol.

[40] Nugent AA, Lin K, van Lengerich B, Lianoglou S, Przybyla L, Davis SS, Llapashtica C, Wang J, Kim DJ, Xia D, Lucas A, Baskaran S, Haddick PCG, Lenser M, Earr TK, Shi J, Dugas JC, Andreone BJ, Logan T, Solanoy HO, Chen H, Srivastava A, Poda SB, Sanchez PE, Watts RJ, Sandmann T, Astarita G, Lewcock JW, Monroe KM, Di Paolo G (2020) TREM2 regulates microglial cholesterol metabolism upon chronic phagocytic challenge. Neuron 105:837 – 54 e9. 10.1016/j.neuron.2019.12.007 10.1016/j.neuron.2019.12.007 31902528

[41] Cantuti-Castelvetri L, Fitzner D, Bosch-Queralt M, Weil MT, Su M, Sen P, Ruhwedel T, Mitkovski M, Trendelenburg G, Lutjohann D, Mobius W, Simons M (2018). Defective cholesterol clearance limits remyelination in the aged central nervous system. Science.

[42] Schmitt S, Castelvetri LC, Simons M (2015). Metabolism and functions of lipids in myelin. Biochim Biophys Acta.

[43] Zhou X, Paushter DH, Pagan MD, Kim D, Nunez Santos M, Lieberman RL, Overkleeft HS, Sun Y, Smolka MB, Hu F (2019). Progranulin deficiency leads to reduced glucocerebrosidase activity. PLoS ONE.

[44] Arrant AE, Roth JR, Boyle NR, Kashyap SN, Hoffmann MQ, Murchison CF, Ramos EM, Nana AL, Spina S, Grinberg LT, Miller BL, Seeley WW, Roberson ED (2019). Impaired ß-glucocerebrosidase activity and processing in frontotemporal dementia due to progranulin mutations. Acta Neuropathol Commun.

[45] Boland S, Swarup S, Ambaw YA, Malia PC, Richards RC, Fischer AW, Singh S, Aggarwal G, Spina S, Nana AL, Grinberg LT, Seeley WW, Surma MA, Klose C, Paulo JA, Nguyen AD, Harper JW, Walther TC, Farese RV (2022). Deficiency of the frontotemporal dementia gene GRN results in gangliosidosis. Nat Commun.

[46] Wang Y, Cella M, Mallinson K, Ulrich JD, Young KL, Robinette ML, Gilfillan S, Krishnan GM, Sudhakar S, Zinselmeyer BH, Holtzman DM, Cirrito JR, Colonna M (2015). TREM2 lipid sensing sustains the microglial response in an Alzheimer’s disease model. Cell.

[47] Woollacott IOC, Bocchetta M, Sudre CH, Ridha BH, Strand C, Courtney R, Ourselin S, Cardoso MJ, Warren JD, Rossor MN, Revesz T, Fox NC, Holton JL, Lashley T, Rohrer JD (2018). Pathological correlates of white matter hyperintensities in a case of progranulin mutation associated frontotemporal dementia. Neurocase.

[48] Sirisi S, Querol-Vilaseca M, Dols-Icardo O, Pegueroles J, Montal V, Munoz L, Torres S, Ferrer-Raventos P, Iulita MF, Sanchez-Aced E, Blesa R, Illan-Gala I, Molina-Porcel L, Borrego-Ecija S, Sanchez-Valle R, Clarimon J, Belbin O, Fortea J, Lleo A (2022). Myelin loss in C9orf72 hexanucleotide expansion carriers. J Neurosci Res.

[49] Wender M, Filipek-Wender H, Stanislawska J (1974). Cholesteryl esters of the brain in demyelinating diseases. Clin Chim Acta.

[50] Theda C, Moser AB, Powers JM, Moser HW (1992). Phospholipids in X-linked adrenoleukodystrophy white matter: fatty acid abnormalities before the onset of demyelination. J Neurol Sci.

[51] Chiang JYL, Ferrell JM (2020). Up to date on cholesterol 7 alpha-hydroxylase (CYP7A1) in bile acid synthesis. Liver Res.

[52] Cantoni C, Bollman B, Licastro D, Xie M, Mikesell R, Schmidt R, Yuede CM, Galimberti D, Olivecrona G, Klein RS, Cross AH, Otero K, Piccio L (2015). TREM2 regulates microglial cell activation in response to demyelination in vivo. Acta Neuropathol.

[53] Wu Y, Shao W, Todd TW, Tong J, Yue M, Koga S, Castanedes-Casey M, Librero AL, Lee CW, Mackenzie IR, Dickson DW, Zhang YJ, Petrucelli L, Prudencio M (2021). Microglial lysosome dysfunction contributes to white matter pathology and TDP-43 proteinopathy in GRN-associated FTD. Cell Rep.

[54] van der Kant R, Langness VF, Herrera CM, Williams DA, Fong LK, Leestemaker Y, Steenvoorden E, Rynearson KD, Brouwers JF, Helms JB, Ovaa H, Giera M, Wagner SL, Bang AG, Goldstein LSB (2019) Cholesterol Metabolism Is a Druggable Axis that Independently Regulates Tau and Amyloid-beta in iPSC-Derived Alzheimer’s Disease Neurons. Cell Stem Cell 24:363 – 75 e9. 10.1016/j.stem.2018.12.013 10.1016/j.stem.2018.12.013 PMC6414424 30686764

[55] Puglielli L, Konopka G, Pack-Chung E, Ingano LA, Berezovska O, Hyman BT, Chang TY, Tanzi RE, Kovacs DM (2001). Acyl-coenzyme A: cholesterol acyltransferase modulates the generation of the amyloid beta-peptide. Nat Cell Biol.

[56] Rinaldo P, Matern D, Bennett MJ (2002). Fatty acid Oxidation Disorders. Annu Rev Physiol.

[57] Jacova C, Hsiung G-YR, Tawankanjanachot I, Dinelle K, McCormick S, Gonzalez M, Lee H, Sengdy P, Bouchard-Kerr P, Baker M, Rademakers R, Sossi V, Stoessl AJ, Feldman HH, Mackenzie IR (2013). Anterior brain glucose hypometabolism predates dementia in progranulin mutation carriers. Neurology.

[58] Turk BR, Theda C, Fatemi A, Moser AB (2020). X-linked adrenoleukodystrophy: Pathology, pathophysiology, diagnostic testing, newborn screening and therapies. Int J Dev neuroscience: official J Int Soc Dev Neurosci.

[59] Viader A, Sasaki Y, Kim S, Strickland A, Workman Cayce S, Yang K, Gross Richard W, Milbrandt J (2013). Aberrant Schwann cell lipid metabolism linked to mitochondrial deficits leads to Axon Degeneration and Neuropathy. Neuron.

[60] Gotzl JK, Brendel M, Werner G, Parhizkar S, Sebastian Monasor L, Kleinberger G, Colombo AV, Deussing M, Wagner M, Winkelmann J, Diehl-Schmid J, Levin J, Fellerer K, Reifschneider A, Bultmann S, Bartenstein P, Rominger A, Tahirovic S, Smith ST, Madore C, Butovsky O, Capell A, Haass C (2019) Opposite microglial activation stages upon loss of PGRN or TREM2 result in reduced cerebral glucose metabolism. EMBO Mol Med 11. 10.15252/emmm.201809711 10.15252/emmm.201809711 PMC6554672 31122931

[61] Lall D, Lorenzini I, Mota TA, Bell S, Mahan TE, Ulrich JD, Davtyan H, Rexach JE, Muhammad A, Shelest O, Landeros J, Vazquez M, Kim J, Ghaffari L, O’Rourke JG, Geschwind DH, Blurton-Jones M, Holtzman DM, Sattler R, Baloh RH (2021) C9orf72 deficiency promotes microglial-mediated synaptic loss in aging and amyloid accumulation. Neuron 109:2275-91 e8. 10.1016/j.neuron.2021.05.020 10.1016/j.neuron.2021.05.020 PMC8298293 34133945

[62] Zhang J, Velmeshev D, Hashimoto K, Huang YH, Hofmann JW, Shi X, Chen J, Leidal AM, Dishart JG, Cahill MK, Kelley KW, Liddelow SA, Seeley WW, Miller BL, Walther TC, Farese RV, Taylor JP, Ullian EM, Huang B, Debnath J, Wittmann T, Kriegstein AR, Huang EJ (2020). Neurotoxic microglia promote TDP-43 proteinopathy in progranulin deficiency. Nature.

[63] O’Rourke JG, Bogdanik L, Yanez A, Lall D, Wolf AJ, Muhammad AK, Ho R, Carmona S, Vit JP, Zarrow J, Kim KJ, Bell S, Harms MB, Miller TM, Dangler CA, Underhill DM, Goodridge HS, Lutz CM, Baloh RH (2016). C9orf72 is required for proper macrophage and microglial function in mice. Science.

[64] Brettschneider J, Toledo JB, Van Deerlin VM, Elman L, McCluskey L, Lee VM, Trojanowski JQ (2012). Microglial activation correlates with disease progression and upper motor neuron clinical symptoms in amyotrophic lateral sclerosis. PLoS ONE.

[65] Sadler GL, Lewis KN, Narayana VK, De Souza DP, Mason J, McLean C, Gonsalvez DG, Turner BJ, Barton SK (2022) Lipid Metabolism Is Dysregulated in the Motor Cortex White Matter in Amyotrophic Lateral Sclerosis. Metabolites 12. 10.3390/metabo12060554 10.3390/metabo12060554 PMC9230865 35736487

[66] Don AS, Hsiao JH, Bleasel JM, Couttas TA, Halliday GM, Kim W (2014). Altered lipid levels provide evidence for myelin dysfunction in multiple system atrophy. Acta Neuropathol Commun.

[67] Xicoy H, Brouwers JF, Wieringa B, Martens GJM (2020) Explorative combined lipid and transcriptomic profiling of Substantia Nigra and Putamen in Parkinson’s Disease. Cells 9. 10.3390/cells9091966 10.3390/cells9091966 PMC7564986 32858884

[68] Wood PL, Tippireddy S, Feriante J, Woltjer RL (2018). Augmented frontal cortex diacylglycerol levels in Parkinson’s disease and Lewy Body Disease. PLoS ONE.

[69] Cheng D, Jenner AM, Shui G, Cheong WF, Mitchell TW, Nealon JR, Kim WS, McCann H, Wenk MR, Halliday GM, Garner B (2011). Lipid pathway alterations in Parkinson’s disease primary visual cortex. PLoS ONE.

[70] Couttas TA, Kain N, Suchowerska AK, Quek LE, Turner N, Fath T, Garner B, Don AS (2016). Loss of ceramide synthase 2 activity, necessary for myelin biosynthesis, precedes tau pathology in the cortical pathogenesis of Alzheimer’s disease. Neurobiol Aging.

[71] Huynh K, Piguet O, Kwok J, Dobson-Stone C, Halliday GM, Hodges JR, Landin-Romero R (2021). Clinical and biological Correlates of White Matter Hyperintensities in patients with behavioral-variant Frontotemporal Dementia and Alzheimer Disease. Neurology.

[72] Mahoney CJ, Ridgway GR, Malone IB, Downey LE, Beck J, Kinnunen KM, Schmitz N, Golden HL, Rohrer JD, Schott JM, Rossor MN, Ourselin S, Mead S, Fox NC, Warren JD (2014). Profiles of white matter tract pathology in frontotemporal dementia. Hum Brain Mapp.

[73] Lam BY, Halliday GM, Irish M, Hodges JR, Piguet O (2014). Longitudinal white matter changes in frontotemporal dementia subtypes. Hum Brain Mapp.

[74] Lok HC, Kwok JB (2021) The role of White Matter Dysfunction and Leukoencephalopathy/Leukodystrophy genes in the aetiology of Frontotemporal Dementias: implications for Novel Approaches to therapeutics. Int J Mol Sci 22. 10.3390/ijms22052541 10.3390/ijms22052541 PMC7961524 33802612

[75] Sud M, Fahy E, Cotter D, Azam K, Vadivelu I, Burant C, Edison A, Fiehn O, Higashi R, Nair KS, Sumner S, Subramaniam S (2016). Metabolomics Workbench: an international repository for metabolomics data and metadata, metabolite standards, protocols, tutorials and training, and analysis tools. Nucleic Acids Res.

