# Supplementary material for: Disrupted myelin lipid metabolism differentiates frontotemporal dementia caused by GRN and C9orf72 gene mutations
Source: Acta Neuropathol Commun. 2023 Mar 27;11:52. doi: 10.1186/s40478-023-01544-7 (PMC10041703; doi:10.1186/s40478-023-01544-7)
Supplement: Supplementary file 2 — Supplementary Table 1. Lipid Class Totals (nmoles/mg protein) in frontal white matter and one-way ANOVA results. BH FDR: Benjamini-Hochberg false discovery rate-corrected p value (q value). P values for Tukey’s post-test are also given. Supplementary Table 2. Lipid Class Totals (nmoles/mg protein) in frontal grey matter and one-way ANOVA results. BH FDR: Benjamini-Hochberg false discovery rate-corrected p value (q value). P values for Tukey’s post-test are also given. Supplementary Table 3. Lipid Class Totals (nmoles/mg protein) in parietal white matter and one-way ANOVA results. BH FDR: Benjamini-Hochberg false discovery rate-corrected p value (q value). P values for Tukey’s post-test are also given. Supplementary Table 4. Lipid Class Totals (nmoles/mg protein) in parietal grey matter and one-way ANOVA results. BH FDR: Benjamini-Hochberg false discovery rate-corrected p value (q value). P values for Tukey’s post-test are also given. [file 40478_2023_1544_MOESM2_ESM.docx]

| **Frontal WM** | Control | | *C9orf72* | | *GRN* | | ANOVA | | BH FDR | Tukey's Posthoc | | |
| --- | --- | --- | --- | --- | --- | --- | --- | --- | --- | --- | --- | --- |
|  | Mean | SD | Mean | SD | Mean | SD | F | *p* | *q* | C9orf72 - Control | GRN - Control | GRN-C9orf72 |
| MAG | 6.500 | 1.733 | 6.224 | 2.759 | 6.839 | 2.549 | 0.268 | 0.767 | 0.796 | 0.963 | 0.867 | 0.747 |
| DAG | 10.149 | 2.591 | 8.152 | 1.069 | 9.185 | 1.120 | 5.639 | 0.010 | 0.027 | 0.007 | 0.448 | 0.258 |
| TAG | 6.538 | 7.604 | 6.101 | 2.870 | 5.302 | 2.064 | 0.359 | 0.702 | 0.758 | 0.691 | 0.860 | 0.983 |
| PC | 305.913 | 430.127 | 219.826 | 114.223 | 128.296 | 28.640 | 0.638 | 0.537 | 0.604 | 0.773 | 0.860 | 0.520 |
| PE(P) | 459.701 | 330.009 | 347.676 | 109.375 | 225.146 | 32.001 | 1.570 | 0.230 | 0.324 | 1.000 | 0.259 | 0.267 |
| LPC | 2.100 | 0.605 | 1.539 | 0.451 | 1.437 | 0.265 | 6.175 | 0.007 | 0.025 | 0.021 | 0.016 | 0.845 |
| LPC(O) | 0.127 | 0.035 | 0.082 | 0.047 | 0.068 | 0.021 | 6.711 | 0.005 | 0.025 | 0.017 | 0.011 | 0.802 |
| AcCa | 0.051 | 0.045 | 0.031 | 0.017 | 0.041 | 0.012 | 1.907 | 0.171 | 0.272 | 0.159 | 0.907 | 0.465 |
| Cholesterol | 1.746 | 2.331 | 1.019 | 0.721 | 0.444 | 0.130 | 1.196 | 0.320 | 0.376 | 1.000 | 0.356 | 0.356 |
| CholE | 3.553 | 3.064 | 4.848 | 4.007 | 10.742 | 4.869 | 4.208 | 0.028 | 0.062 | 0.725 | 0.024 | 0.091 |
| Hex2Cer | 196.320 | 93.300 | 112.553 | 49.489 | 75.041 | 22.949 | 9.845 | 0.001 | 0.011 | 0.015 | 0.001 | 0.253 |
| Sph | 0.087 | 0.066 | 0.163 | 0.128 | 0.119 | 0.073 | 1.414 | 0.264 | 0.324 | 0.238 | 0.639 | 0.869 |
| SM | 412.869 | 286.549 | 196.942 | 70.461 | 155.417 | 32.340 | 8.095 | 0.002 | 0.015 | 0.009 | 0.005 | 0.767 |
| Sulfatide | 83.803 | 42.150 | 45.809 | 13.315 | 35.136 | 6.835 | 12.129 | 0.000 | 0.007 | 0.003 | 0.000 | 0.441 |
| Hex1Cer | 803.618 | 763.146 | 290.641 | 111.876 | 235.188 | 66.833 | 5.923 | 0.008 | 0.025 | 0.022 | 0.019 | 0.874 |
| PE | 146.829 | 86.858 | 120.708 | 45.517 | 67.545 | 14.819 | 4.568 | 0.021 | 0.052 | 0.828 | 0.019 | 0.057 |
| PE (O) | 411.505 | 353.807 | 321.152 | 131.002 | 157.318 | 37.697 | 3.891 | 0.035 | 0.073 | 0.991 | 0.042 | 0.054 |
| LPE | 2.471 | 1.410 | 1.601 | 0.532 | 1.813 | 0.583 | 2.777 | 0.083 | 0.150 | 0.070 | 0.438 | 0.741 |
| LPE(O) | 1.016 | 0.256 | 0.837 | 0.370 | 0.807 | 0.181 | 2.027 | 0.155 | 0.261 | 0.177 | 0.303 | 0.998 |
| LPA | 0.127 | 0.045 | 0.105 | 0.015 | 0.105 | 0.009 | 1.514 | 0.241 | 0.324 | 0.434 | 0.255 | 0.842 |
| LPS | 0.352 | 0.094 | 0.309 | 0.082 | 0.315 | 0.061 | 1.437 | 0.258 | 0.324 | 0.299 | 0.396 | 0.999 |
| LPI | 0.271 | 0.126 | 0.185 | 0.051 | 0.118 | 0.030 | 8.174 | 0.002 | 0.015 | 0.214 | 0.001 | 0.043 |
| PG | 1.388 | 0.449 | 1.195 | 0.287 | 1.091 | 0.189 | 1.539 | 0.236 | 0.324 | 0.564 | 0.217 | 0.678 |
| PI | 21.293 | 6.459 | 16.717 | 2.330 | 14.093 | 1.996 | 6.264 | 0.007 | 0.025 | 0.085 | 0.006 | 0.296 |
| PS | 421.003 | 276.429 | 287.022 | 92.876 | 197.901 | 62.429 | 3.155 | 0.062 | 0.119 | 0.602 | 0.049 | 0.231 |
| CL | 0.092 | 0.028 | 0.096 | 0.040 | 0.092 | 0.056 | 0.071 | 0.932 | 0.932 | 0.998 | 0.930 | 0.947 |
| Cer | 9.554 | 5.259 | 5.954 | 2.552 | 3.783 | 1.451 | 6.001 | 0.008 | 0.025 | 0.111 | 0.007 | 0.266 |

**Supplementary Table 1.** Lipid Class Totals (nmoles/mg protein) in frontal white matter and one-way ANOVA results. BH FDR: Benjamini-Hochberg false discovery rate-corrected *p* value (*q* value). P values for Tukey’s post-test are also given.

| **Frontal GM** | Control | | *C9orf72* | | *GRN* | | ANOVA | | BH FDR | Tukey's Posthoc | | |
| --- | --- | --- | --- | --- | --- | --- | --- | --- | --- | --- | --- | --- |
|  | Mean | SD | Mean | SD | Mean | SD | F | *p* | *q* | C9orf72 - Control | GRN - Control | GRN-C9orf72 |
| MAG | 20.814 | 15.208 | 15.313 | 11.727 | 31.857 | 11.889 | 2.419 | 0.111 | 0.334 | 0.851 | 0.225 | 0.099 |
| DAG | 7.756 | 3.713 | 7.891 | 2.453 | 11.469 | 4.609 | 3.002 | 0.069 | 0.268 | 0.579 | 0.055 | 0.268 |
| TAG | 21.821 | 13.673 | 20.350 | 13.536 | 43.373 | 35.244 | 1.349 | 0.279 | 0.539 | 0.985 | 0.286 | 0.352 |
| PC | 476.001 | 194.446 | 454.667 | 220.448 | 514.049 | 230.176 | 0.208 | 0.814 | 0.916 | 0.971 | 0.896 | 0.798 |
| PE(P) | 242.192 | 76.110 | 273.771 | 110.688 | 244.143 | 113.074 | 0.312 | 0.735 | 0.911 | 0.725 | 0.987 | 0.874 |
| LPC | 2.546 | 1.013 | 2.349 | 0.802 | 2.255 | 0.654 | 0.116 | 0.891 | 0.925 | 0.946 | 0.889 | 0.980 |
| LPC(O) | 0.022 | 0.007 | 0.029 | 0.014 | 0.037 | 0.009 | 3.629 | 0.043 | 0.249 | 0.309 | 0.035 | 0.362 |
| AcCa | 0.026 | 0.015 | 0.028 | 0.014 | 0.063 | 0.014 | 8.536 | 0.002 | 0.046 | 0.931 | 0.002 | 0.004 |
| Cholesterol | 0.578 | 0.330 | 0.765 | 0.776 | 0.862 | 0.952 | 0.276 | 0.762 | 0.911 | 0.933 | 0.741 | 0.899 |
| CholE | 4.879 | 3.125 | 6.612 | 8.042 | 9.515 | 4.750 | 2.194 | 0.134 | 0.347 | 0.997 | 0.171 | 0.153 |
| Hex2Cer | 15.196 | 9.916 | 27.554 | 21.271 | 14.203 | 6.327 | 2.111 | 0.144 | 0.347 | 0.142 | 0.953 | 0.369 |
| Sph | 0.084 | 0.057 | 0.131 | 0.162 | 0.305 | 0.266 | 3.947 | 0.034 | 0.249 | 0.693 | 0.027 | 0.115 |
| SM | 69.164 | 19.799 | 88.980 | 34.207 | 74.977 | 16.045 | 1.128 | 0.341 | 0.575 | 0.327 | 0.954 | 0.617 |
| Sulfatide | 10.805 | 5.320 | 15.200 | 8.855 | 8.559 | 3.346 | 1.741 | 0.198 | 0.411 | 0.299 | 0.940 | 0.254 |
| Hex1Cer | 74.992 | 31.038 | 111.736 | 72.774 | 68.025 | 25.529 | 2.028 | 0.154 | 0.347 | 0.206 | 0.987 | 0.248 |
| PE | 124.027 | 50.578 | 120.590 | 56.558 | 127.009 | 60.116 | 0.045 | 0.956 | 0.956 | 0.997 | 0.953 | 0.971 |
| PE (O) | 169.086 | 62.143 | 183.330 | 80.399 | 185.754 | 84.147 | 0.256 | 0.776 | 0.911 | 0.824 | 0.814 | 0.994 |
| LPE | 2.708 | 1.002 | 2.416 | 0.831 | 2.623 | 1.020 | 0.289 | 0.752 | 0.911 | 0.730 | 0.940 | 0.949 |
| LPE(O) | 0.451 | 0.150 | 0.584 | 0.211 | 0.960 | 0.568 | 3.469 | 0.048 | 0.249 | 0.610 | 0.038 | 0.186 |
| LPA | 0.255 | 0.097 | 0.260 | 0.097 | 0.394 | 0.149 | 3.291 | 0.055 | 0.249 | 0.851 | 0.050 | 0.125 |
| LPS | 0.448 | 0.142 | 0.507 | 0.171 | 0.653 | 0.245 | 1.175 | 0.327 | 0.575 | 0.969 | 0.320 | 0.425 |
| LPI | 0.288 | 0.084 | 0.300 | 0.091 | 0.170 | 0.056 | 5.104 | 0.015 | 0.198 | 0.883 | 0.036 | 0.015 |
| PG | 2.355 | 1.044 | 2.438 | 0.915 | 2.619 | 1.238 | 0.291 | 0.750 | 0.911 | 0.833 | 0.767 | 0.977 |
| PI | 30.036 | 12.520 | 29.860 | 10.169 | 28.568 | 10.596 | 0.143 | 0.868 | 0.925 | 0.855 | 0.969 | 0.975 |
| PS | 238.961 | 71.907 | 260.720 | 79.399 | 241.059 | 75.789 | 0.303 | 0.742 | 0.911 | 0.727 | 0.894 | 0.978 |
| CL | 0.573 | 0.163 | 0.624 | 0.205 | 0.482 | 0.179 | 1.007 | 0.381 | 0.605 | 0.979 | 0.470 | 0.379 |
| Cer | 1.681 | 0.507 | 2.371 | 0.958 | 2.581 | 0.636 | 2.637 | 0.093 | 0.314 | 0.197 | 0.119 | 0.846 |

**Supplementary Table 2.** Lipid Class Totals (nmoles/mg protein) in frontal grey matter and one-way ANOVA results. BH FDR: Benjamini-Hochberg false discovery rate-corrected *p* value (*q* value). P values for Tukey’s post-test are also given.

| **Parietal WM** | Control | | *C9orf72* | | *GRN* | | ANOVA | | BH FDR | Tukey's Posthoc | | |
| --- | --- | --- | --- | --- | --- | --- | --- | --- | --- | --- | --- | --- |
|  | Mean | SD | Mean | SD | Mean | SD | F | *p* | *q* | C9orf72 - Control | GRN - Control | GRN-C9orf72 |
| MAG | 12.571 | 5.527 | 14.200 | 7.235 | 17.703 | 11.690 | 0.410 | 0.668 | 0.784 | 1.000 | 0.697 | 0.690 |
| DAG | 17.712 | 6.095 | 19.079 | 2.697 | 19.591 | 5.981 | 0.600 | 0.557 | 0.715 | 0.804 | 0.536 | 0.851 |
| TAG | 8.147 | 5.294 | 5.584 | 1.268 | 5.852 | 3.461 | 2.224 | 0.131 | 0.442 | 0.138 | 0.313 | 0.979 |
| PC | 343.264 | 401.649 | 210.167 | 126.093 | 209.059 | 102.045 | 0.214 | 0.809 | 0.874 | 0.883 | 0.817 | 0.977 |
| PE(P) | 439.224 | 241.152 | 357.976 | 96.042 | 318.190 | 120.959 | 1.233 | 0.310 | 0.558 | 0.730 | 0.279 | 0.632 |
| LPC | 2.996 | 0.281 | 2.733 | 0.436 | 2.921 | 0.494 | 0.630 | 0.541 | 0.715 | 0.509 | 0.866 | 0.898 |
| LPC(O) | 0.183 | 0.054 | 0.149 | 0.047 | 0.219 | 0.086 | 1.097 | 0.351 | 0.592 | 0.574 | 0.847 | 0.352 |
| AcCa | 0.077 | 0.062 | 0.066 | 0.074 | 0.082 | 0.027 | 0.889 | 0.425 | 0.674 | 0.508 | 0.983 | 0.506 |
| Cholesterol | 2.538 | 3.871 | 0.591 | 0.486 | 0.582 | 0.313 | 1.444 | 0.257 | 0.495 | 0.379 | 0.307 | 0.934 |
| CholE | 6.377 | 8.682 | 2.964 | 1.129 | 35.569 | 34.919 | 11.147 | 0.000 | 0.007 | 0.930 | 0.001 | 0.001 |
| Hex2Cer | 290.477 | 81.699 | 264.025 | 42.861 | 241.910 | 66.842 | 1.660 | 0.212 | 0.495 | 0.995 | 0.230 | 0.262 |
| Sph | 0.114 | 0.058 | 0.180 | 0.100 | 0.512 | 0.451 | 10.688 | 0.001 | 0.007 | 0.295 | 0.000 | 0.008 |
| SM | 596.699 | 238.916 | 546.332 | 140.218 | 541.386 | 176.114 | 0.064 | 0.938 | 0.975 | 0.982 | 0.978 | 0.933 |
| Sulfatide | 117.075 | 26.990 | 111.581 | 22.034 | 103.562 | 21.117 | 0.553 | 0.583 | 0.715 | 0.984 | 0.665 | 0.575 |
| Hex1Cer | 1128.162 | 824.735 | 931.701 | 422.655 | 989.405 | 640.833 | 0.022 | 0.978 | 0.978 | 0.988 | 0.978 | 0.997 |
| PE | 207.603 | 92.394 | 154.010 | 49.120 | 157.579 | 36.339 | 1.944 | 0.166 | 0.448 | 0.217 | 0.266 | 0.991 |
| PE (O) | 587.527 | 492.504 | 395.876 | 171.146 | 407.898 | 95.054 | 0.576 | 0.570 | 0.715 | 0.656 | 0.627 | 0.982 |
| LPE | 2.985 | 0.528 | 3.404 | 1.358 | 5.304 | 1.700 | 6.874 | 0.005 | 0.041 | 0.763 | 0.004 | 0.017 |
| LPE(O) | 1.507 | 0.233 | 1.414 | 0.265 | 1.899 | 0.604 | 2.063 | 0.150 | 0.448 | 0.832 | 0.299 | 0.132 |
| LPA | 0.185 | 0.026 | 0.162 | 0.018 | 0.166 | 0.035 | 2.779 | 0.083 | 0.320 | 0.100 | 0.200 | 0.998 |
| LPS | 0.560 | 0.106 | 0.593 | 0.084 | 0.757 | 0.132 | 5.284 | 0.013 | 0.087 | 0.544 | 0.010 | 0.071 |
| LPI | 0.276 | 0.071 | 0.230 | 0.046 | 0.185 | 0.055 | 4.798 | 0.018 | 0.098 | 0.630 | 0.014 | 0.078 |
| PG | 2.724 | 0.920 | 2.131 | 0.334 | 1.977 | 1.032 | 3.231 | 0.058 | 0.261 | 0.152 | 0.073 | 0.780 |
| PI | 30.770 | 3.499 | 29.829 | 2.633 | 27.441 | 5.057 | 1.552 | 0.233 | 0.495 | 0.937 | 0.222 | 0.347 |
| PS | 436.151 | 119.021 | 431.349 | 103.793 | 404.113 | 55.046 | 0.577 | 0.570 | 0.715 | 1.000 | 0.602 | 0.597 |
| CL | 0.127 | 0.043 | 0.133 | 0.032 | 0.116 | 0.033 | 0.282 | 0.757 | 0.851 | 0.911 | 0.917 | 0.739 |
| Cer | 11.240 | 3.106 | 10.671 | 2.774 | 8.410 | 2.561 | 1.459 | 0.253 | 0.495 | 0.970 | 0.252 | 0.340 |

**Supplementary Table 3.** Lipid Class Totals (nmoles/mg protein) in parietal white matter and one-way ANOVA results. BH FDR: Benjamini-Hochberg false discovery rate-corrected *p* value (*q* value). P values for Tukey’s post-test are also given.

| **Parietal GM** | Control | | *C9orf72* | | *GRN* | | ANOVA | | BH FDR | Tukey's Posthoc | | |
| --- | --- | --- | --- | --- | --- | --- | --- | --- | --- | --- | --- | --- |
|  | Mean | SD | Mean | SD | Mean | SD | F | *p* | *q* | C9orf72 - Control | GRN - Control | GRN-C9orf72 |
| MAG | 23.995 | 17.776 | 13.593 | 6.075 | 10.198 | 3.837 | 3.307 | 0.055 | 0.738 | 0.131 | 0.075 | 0.823 |
| DAG | 10.582 | 3.678 | 10.039 | 3.993 | 8.285 | 1.851 | 0.573 | 0.572 | 0.978 | 0.944 | 0.545 | 0.714 |
| TAG | 26.265 | 17.619 | 28.680 | 34.300 | 18.249 | 14.978 | 0.271 | 0.765 | 0.978 | 0.976 | 0.749 | 0.848 |
| PC | 365.620 | 158.491 | 300.347 | 87.681 | 324.438 | 72.914 | 0.171 | 0.844 | 0.978 | 0.849 | 0.999 | 0.907 |
| PE(P) | 211.638 | 44.253 | 198.551 | 54.407 | 206.041 | 44.089 | 0.057 | 0.944 | 0.978 | 0.939 | 0.987 | 0.990 |
| LPC | 2.560 | 0.488 | 2.302 | 0.811 | 2.382 | 0.584 | 0.233 | 0.794 | 0.978 | 0.781 | 0.918 | 0.983 |
| LPC(O) | 0.023 | 0.005 | 0.026 | 0.010 | 0.032 | 0.006 | 2.087 | 0.147 | 0.798 | 0.758 | 0.125 | 0.341 |
| AcCa | 0.036 | 0.026 | 0.037 | 0.020 | 0.060 | 0.022 | 2.604 | 0.096 | 0.798 | 0.945 | 0.095 | 0.158 |
| Cholesterol | 0.785 | 0.696 | 1.586 | 2.072 | 0.953 | 0.638 | 0.585 | 0.565 | 0.978 | 0.554 | 0.773 | 0.977 |
| CholE | 5.064 | 5.361 | 9.270 | 11.099 | 7.350 | 7.194 | 0.812 | 0.456 | 0.978 | 0.473 | 0.628 | 0.997 |
| Hex2Cer | 20.168 | 12.134 | 23.581 | 11.974 | 22.234 | 13.581 | 0.039 | 0.961 | 0.978 | 0.958 | 0.997 | 0.986 |
| Sph | 0.111 | 0.072 | 0.165 | 0.209 | 0.082 | 0.050 | 0.689 | 0.512 | 0.978 | 0.891 | 0.714 | 0.479 |
| SM | 66.811 | 16.731 | 79.444 | 28.445 | 84.035 | 17.031 | 0.852 | 0.440 | 0.978 | 0.621 | 0.453 | 0.907 |
| Sulfatide | 15.892 | 8.291 | 18.837 | 9.110 | 18.894 | 11.108 | 0.057 | 0.945 | 0.978 | 0.940 | 0.981 | 0.995 |
| Hex1Cer | 120.270 | 49.881 | 141.471 | 68.912 | 115.772 | 50.087 | 0.144 | 0.867 | 0.978 | 0.929 | 0.978 | 0.866 |
| PE | 120.475 | 43.315 | 99.780 | 32.438 | 97.589 | 14.897 | 0.628 | 0.543 | 0.978 | 0.595 | 0.640 | 0.997 |
| PE (O) | 163.872 | 41.842 | 148.853 | 44.933 | 153.569 | 34.995 | 0.142 | 0.868 | 0.978 | 0.861 | 0.947 | 0.991 |
| LPE | 2.788 | 0.504 | 2.663 | 0.800 | 3.817 | 1.410 | 1.778 | 0.191 | 0.861 | 0.857 | 0.348 | 0.171 |
| LPE(O) | 0.421 | 0.123 | 0.442 | 0.142 | 0.850 | 0.385 | 6.564 | 0.006 | 0.150 | 0.997 | 0.009 | 0.008 |
| LPA | 0.237 | 0.088 | 0.223 | 0.103 | 0.233 | 0.030 | 0.251 | 0.780 | 0.978 | 0.960 | 0.881 | 0.760 |
| LPS | 0.621 | 0.175 | 0.633 | 0.161 | 0.859 | 0.279 | 2.081 | 0.148 | 0.798 | 0.996 | 0.188 | 0.166 |
| LPI | 0.224 | 0.047 | 0.236 | 0.076 | 0.187 | 0.039 | 1.061 | 0.362 | 0.978 | 0.867 | 0.570 | 0.331 |
| PG | 2.556 | 0.543 | 2.445 | 0.957 | 2.033 | 0.268 | 0.681 | 0.516 | 0.978 | 0.953 | 0.494 | 0.647 |
| PI | 33.521 | 6.833 | 33.254 | 10.082 | 31.251 | 4.110 | 0.023 | 0.978 | 0.978 | 1.000 | 0.982 | 0.977 |
| PS | 227.613 | 43.411 | 227.145 | 57.718 | 234.499 | 22.370 | 0.151 | 0.861 | 0.978 | 0.980 | 0.848 | 0.922 |
| CL | 0.483 | 0.110 | 0.439 | 0.102 | 0.410 | 0.086 | 0.475 | 0.628 | 0.978 | 0.757 | 0.642 | 0.952 |
| Cer | 2.211 | 0.596 | 2.597 | 1.068 | 3.101 | 0.786 | 1.380 | 0.272 | 0.978 | 0.898 | 0.249 | 0.429 |

**Supplementary Table 4.** Lipid Class Totals (nmoles/mg protein) in parietal grey matter and one-way ANOVA results. BH FDR: Benjamini-Hochberg false discovery rate-corrected *p* value (*q* value). P values for Tukey’s post-test are also given.
